# The efficacy of early prone or lateral positioning in severe COVID-19 patients: A single-center prospective cohort

**DOI:** 10.1093/pcmedi/pbaa034

**Published:** 2020-09-28

**Authors:** Zhong Ni, Kaige Wang, Ting Wang, Yuenan Ni, Wei Huang, Ping Zhu, Tao Fan, Ye Wang, Bo Wang, Jun Deng, Zhicheng Qian, Jiasheng Liu, Wenhao Cai, Shanling Xu, Yu Du, Gang Wang, Zongan Liang, Weimin Li, Jianfei Luo, Fengming Luo, Dan Liu

**Affiliations:** Department of Respiratory and Critical Care Medicine, Clinical Research Center for Respiratory Disease, West China Hospital, Sichuan University, Chengdu, China; Department of Respiratory and Critical Care Medicine, Clinical Research Center for Respiratory Disease, West China Hospital, Sichuan University, Chengdu, China; Department of Respiratory and Critical Care Medicine, Clinical Research Center for Respiratory Disease, West China Hospital, Sichuan University, Chengdu, China; Department of Respiratory and Critical Care Medicine, Clinical Research Center for Respiratory Disease, West China Hospital, Sichuan University, Chengdu, China; Department of Integrated Traditional Chinese and Western Medicine, West China Hospital, Sichuan University, Chengdu, China; Department of Integrated Traditional Chinese and Western Medicine, West China Hospital, Sichuan University, Chengdu, China; Department of Integrated Traditional Chinese and Western Medicine, West China Hospital, Sichuan University, Chengdu, China; Department of Respiratory and Critical Care Medicine, Clinical Research Center for Respiratory Disease, West China Hospital, Sichuan University, Chengdu, China; Department of Respiratory and Critical Care Medicine, Clinical Research Center for Respiratory Disease, West China Hospital, Sichuan University, Chengdu, China; Department of Respiratory and Critical Care Medicine, The Affiliated Hospital of Southwest Medical University, Luzhou, China; Intensive Care Unit, the Affiliated Hospital of North Sichuan Medical College, Nanchong, China; Department of Gastrointestinal Surgery, Renmin Hospital of Wuhan University, Wuhan, China; Department of Integrated Traditional Chinese and Western Medicine, West China Hospital, Sichuan University, Chengdu, China; Critical Care Medicine Department, Sichuan Cancer Hospital, Affiliated Cancer Hospital to University of Electronic Science and Technology of China, Chengdu, China; Department of Emergency and Critical Care Medicine, West China School of Public Health, West China Fourth Hospital, Sichuan University, Chengdu, China; Department of Respiratory and Critical Care Medicine, Clinical Research Center for Respiratory Disease, West China Hospital, Sichuan University, Chengdu, China; Department of Respiratory and Critical Care Medicine, Clinical Research Center for Respiratory Disease, West China Hospital, Sichuan University, Chengdu, China; Department of Respiratory and Critical Care Medicine, Clinical Research Center for Respiratory Disease, West China Hospital, Sichuan University, Chengdu, China; Department of Gastrointestinal Surgery, Renmin Hospital of Wuhan University, Wuhan, China; Department of Respiratory and Critical Care Medicine, Clinical Research Center for Respiratory Disease, West China Hospital, Sichuan University, Chengdu, China; Department of Respiratory and Critical Care Medicine, Clinical Research Center for Respiratory Disease, West China Hospital, Sichuan University, Chengdu, China

## Abstract

**Background:**

Position intervention has been shown to improve oxygenation, but its role in non-invasively ventilated severe COVID-19 patients has never been assessed. The objective of this study was to investigate the efficacy of early position intervention on non-invasively ventilated severe COVID-19 patients.

**Methods:**

This was a single-center, prospective observational study in consecutive severe COVID-19 patients managed in a provisional ICU at Renmin Hospital of Wuhan University during January 31^st^ to February 15^th^, 2020. Patients with chest CT showing exudation or consolidation in bilateral peripheral and posterior part of the lungs were included. Early position intervention (prone or lateral) was commenced for > 4 hours daily for 10 days, while others had standard care.

**Results:**

The baseline parameters were comparable between position intervention group (*n* = 17) and standard care group (*n* = 35). Position intervention was well-tolerated and increased cumulative adjusted mean difference of SpO_2_/FiO_2_ (409, 95% CI 86 to 733) and ROX index (26, 95% CI 9 to 43) with decreased Borg scale (−9, 95 CI −15 to −3) during first 7 days. It also facilitated absorption of lung lesions and reduced patients of high National Early Warning Score 2 (≥ 7) on days 7 and 14 with a trend toward fastened clinical improvement. Virus shedding and length of hospital stay were comparable between the two groups.

**Conclusions:**

This study provides the first evidence for improved oxygenation and lung lesion absorption using early position intervention in non-invasively ventilated severe COVID-19 patients and warrants further randomized trials.

## Introduction

The coronavirus disease 2019 (COVID-19), characterized by atypical pneumonia, had an outbreak in December 2019 in Wuhan, Central China (1). Now it has caused a global pandemic with increasing incidence, mortality, medical resource consumption, and social-economic burdens. The full spectrum of COVID-19 ranges from mild, self-limiting respiratory tract illness to severe progressive pneumonia, predominately manifesting as acute respiratory distress syndrome (ARDS) that requiring Intensive Care Unit (ICU) admission (2). The rate of invasive mechanical ventilation and mortality of severe COVID-19 patients remains high, partially attributed to delayed admission and intervention during the outbreak (3, 4). Despite several endeavors of randomized, placebo-controlled trial of Lopinavir-Ritonavir (5) and compassionate use of Remdesivir (6), chloroquine (7), convalescent plasma (8), there remains no approved pharmacological treatment with definite efficacy for this devastating disease.

Prone positioning has been shown to improve oxygenation and reduce complications and mortality in moderate-to-severe ARDS patients with invasive mechanical ventilation (9, 10). Likewise, lateral positioning was associated with improved pulmonary gas exchange and drainage of secretions in critically ill patients, albeit its efficacy was inconclusive (11). The latest guidance issued by the National Health Commission (NHC) of China

ORIGINAL UNEDITED MANUSCRIPT

and the World Health Organization (WHO) for COVID-19 management only recommended that prone positioning could be applied in critically ill patients who were under invasive mechanical ventilation. A recent preliminary study of 12 critically ill COVID-19 patients has shown that position invention was associated with improved oxygenation and lung recruitability (12). However, the effects of position intervention have never been assessed in awake severe COVID-19 patients without intubation at admission.

The objective of this study was to investigate the impact of early prone or lateral positioning on oxygenation improvement, lung lesion absorption, and other clinical outcomes in awake severe COVID-19 patients.

**Methods**

**Study Design and Patients**

This prospective, observational cohort study, was designed, conducted and reported according to Strengthening the Reporting of Observational Studies in Epidemiology (STROBE) guidelines (13). Consecutive patients with COVID-19 admitted to provisional ICUs in Renmin Hospital of Wuhan University (between January 31st to February 15th, 2020) were scrutinized for inclusion. This study sought to compare the clinical outcomes between patients who received early position intervention and those who had standard care only. The study protocol was reviewed and approved by the Institutional Review Board of Renmin Hospital of Wuhan University (No. WDRY2020-K068). On a sperate note, studies in China comparing ICU vs. non-ICU or severe vs. non-

ORIGINAL UNEDITED MANUSCRIPT

severe were manually searched (by W.C. to 20th April 2020) to understand the baseline characteristics of these patients.

The diagnosis of COVID-19 was defined as presence of severe acute respiratory syndrome coronavirus 2 before admission, determined by reverse transcription-polymerase chain reaction method as per NHC of China (14) and WHO (15) standards. Patients were included if met the following criteria: classified as severe category of COVID-19, manifesting as dyspnea with the respiratory rate (RR) ≥ 30 breaths/min, pulse oxygen saturation ≤ 93% at rest, or partial pressure of arterial oxygen ($PaO_2$) to fraction of inspired oxygen ($FiO_2$) ratio ≤ 300 mmHg (14); Chest computerized tomographic (CT) images showing exudation or consolidation mainly in the bilateral peripheral and posterior part of the lungs.

Patients were excluded if they were aged < 18 or > 80 years old; were pregnant; were critically ill (invasive mechanical ventilation, severe cardiac failure, or hemodynamically unstable) (14); contraindicated to the prone or lateral position (i.e. a history of the vertebral disease); were unable to cooperate.

Three attending physicians independently securitized eligibility of patients for recruitment (Z.L., J.L., and T.W.). If there was a disagreement, final decision was made by the medical team leader (D.L.).

**Interventions and Measurements**

All patients received standard care according to the interim guidance version 4 issued by the NHC of China (14). According to whether received position intervention, these patients were divided into two groups:

ORIGINAL UNEDITED MANUSCRIPT

1. Standard care group: the standard care comprised supplemental oxygen and ventilation, antivirals (ribavirin or arbidor), antibiotics, anticoagulants, and glucocorticoids, as necessary, based on patients' clinical condition. In this group, no position intervention was introduced.

2. Position intervention group: prone positioning was superimposed on the standard care at doctors' own discretion without pre-defined selection criteria. When the prone positioning was not tolerated by the patients, lateral positioning was implemented as an alternative. Each patient was placed for at least 4 hours per day for 10 days. The exact position placement was performed and managed by an experienced respiratory therapist and a physician, and all the patients were evaluated every morning.

Vital signs, oxygen saturation measured by pulse oximetry ($SpO_2$), $FiO_2$, and Borg scale (a 0-10 category scale verbal description for severity of dyspnea) (16) were recorded between 9 and 11 am daily for at least 7 days. To determine illness severity, the Pneumonia Severity Index (PSI) (17) was estimated within 24 hours of admission. The risk of clinical deterioration were assessed by 3 levels of National Early Warning Score 2 (NEWS2): Low score, an aggregate score of 1-4 (or a score of 3 in any one parameter); Medium score, an aggregate score of 5 or 6, indicative of potential severe acute clinical deterioration and the need for an urgent clinical response; High score, an aggregate score of 7 or more (18). NEWS2 was assessed at enrollment, on days 7 and 14 thereafter or before decreased.

Beside the chest CT scan prior to admission, all patients in this study underwent follow-up CT examinations on day 10 after enrollment. The imaging results were interpreted by two attending radiologists who were

ORIGINAL UNEDITED MANUSCRIPT

unaware of the study design. The principle of discharge was based on relief of symptoms, obvious absorption of inflammation in chest CT, abatement of fever, and viral clearance with throat swabs for two consecutive times more than 24 hours apart.

**Data Collection**

The data collection process followed quality assurance and standard operating procedures developed by senior authors (W.L., J.L., F.L., and D.L.). Two researchers (Z.L. and J.L.) independently started data collection at enrollment from electronic medical records independently using pre-defined *pro forma.* Data quality were checked by a third researcher (T.W.). These data contained epidemiological, virologic, clinical, laboratory, microbiological, radiological characteristics, disease severity indices, respiratory support method, pharmacological treatment, and other management details.

**Outcome and Safety Measures**

The primary outcome was oxygenation improvement, determined by cumulative adjusted mean difference of $SpO_2/FiO_2$ (served as oxygen saturation index), Respiratory rate-Oxygenation (ROX) index, and Borg scale between position intervention and standard care.

Secondary outcomes were lung lesion absorption, NEWS2, time to clinical improvement, rate of intubation avoidance, death, time to virus shredding, length of hospital stay, and adverse events. The lung lesion absorption was described as changes of chest CT manifestations after treatment as contrast to admission imaging findings. A semi-quantitative

ORIGINAL UNEDITED MANUSCRIPT

scoring system was used to quantitatively estimate the pulmonary involvement of all abnormalities on the basis of the area involved (19). Each of the 5 lung lobes was visually scored from 0 to 5 as previous study: 0, no involvement; 1, < 5% involvement; 2, 25% involvement; 3, 26%-49% involvement; 4, 50%-75% involvement; 5, > 75% involvement. The total CT score was the sum of the individual lobar scores and ranged from 0 (no involvement) to 25 (maximum involvement) (20). The changes were stratified into obvious absorption (absorption proportion > 30%) and stable or deterioration (absorption proportion ≤ 30%, without absorption or lesion enlargement).

The time to clinical improvement was defined as the time from enrollment to an improvement of two points on a seven-category ordinal scale or live discharge from the hospital, whichever came first (21). Adverse events for position intervention included hemodynamic instability and pressure sore (9, 22).

**Statistical Analysis**

Continuous variables are presented as mean (SD) or median (IQR), compared by Mann-Whitey *U* test. Categorical variables are expressed as frequency (percentage), compared by Chi-square or Fisher's exact test as appropriate. Logistic regression models were used to generate propensity scores that estimated the odds of receiving position intervention versus standard care, covariates are as age, gender, and heart rate. Adjustment for differences in characteristics on admission was performed using inverse probability of treatment weighting (IPTW) models, to assess the effect of

ORIGINAL UNEDITED MANUSCRIPT

position intervention on assiciation of oxygenation improvement parameters demonstrated as forest plots. The time to clinical improvement was assessed when all patients had reached day 30 from enrollment. Data were censored if patients failed to reach clinical improvement at day 30 or decreased before. Survival analysis was done by Kaplan-Meier estimation (Log-rank test). Intention-to-treat analysis was additionally performed to verify our results.

Statistical analysis was performed using the R statistical computing environment (version 3.63). A two-sided *P* value of 0.05 or less was considered to be statistically significant.

## Results

### Characteristics and Treatments of Included Patients

The patient selection process is described in **Figure 1**. A total of 197 consecutive patients with confirmative diagnosis of COVID-19 were screened for inclusion during the study period. Of these, 142 were considered ineligible due to various of reasons. The remaining 55 severe patients were included, of whom 20 received position intervention in contrast to 35 had standard care. Three patients in the position intervention group dropped out due to incompliance to the procedures. Baseline characteristics of position intervention ($n$ = 17) vs. standard care ($n$ = 35) are compared and summarized in **Table 1**. The mean age was 62 (SD 12) years old with 32 (62%) males, 33 (64%) had comorbid chronic diseases, including hypertension (33%), diabetes (21%), coronary heart disease (14%), chronic obstructive pulmonary disease (12%), hepatitis B (4%), pulmonary embolism (2%), hypothyroidism (2%), cerebral ischemic stroke (2%) and malignancy

ORIGINAL UNEDITED MANUSCRIPT

(2%). The median time from symptom onset to hospital admission was 10 (IQR 7-14) days. At presentation, the mean for respiratory rate, $PaO_2/FiO_2$, PSI were 25 (SD 5), 133 (SD 58), and 99 (SD 20), respectively, and the median for NEWS2 was 8 (IQR 7-9); the medians of lymphocytes (0.67, IQR 0.49-0.93), lactate dehydrogenase (375, IQR 306-479), C-reactive protein (73, IQR 46-120), and D-dimer (1.9, IQR 1.18-4.18) were out of normal range. Chest CT revealed that the predominant pattern of temporal abnormalities was diffuse ground-glass opacity with mainly bilateral and subpleural distributions. These findings are similar to clinical profiles of severe COVID patients in China reported by literature (**Supplementary Tables E1-3**).

There were no significant differences between position intervention group and standard care group in terms of baseline demographics, time to admission, oxygenation status, severity indices, and laboratory markers (**Table 1**). There were also no significant differences for respiratory support, antivirals, antibiotics, anticoagulants, and glucocorticoids (and duration) between the two groups (**Table 2**).

**Outcome Measures**

Of 17 patients receiving position intervention, 11 had prone positioning throughout the study with the remaining 6 were changed to lateral positioning. The trend of changes for $SpO_2/FiO_2$, ROX index, and Borg scale with time grouped by position intervention (prone or lateral) and standard care is shown in **Supplementary figure E1**. Compared to the standard care group, there was a gradual increase in both $SpO_2/FiO_2$ and ROX index with time that was mirrored by a significant decrease of Borg scale in either prone or lateral

ORIGINAL UNEDITED MANUSCRIPT

positioning group. Nevertheless, no significant difference between prone and lateral positioning groups was observed for these parameters.

Treatment effects of position intervention on oxygenation improvement are expressed as mean between-group difference (95% CI) and demonstrated by forest plots (**Figure 2**). The mean difference of $SpO_2/FiO_2$ started to increase significantly since day 4 with a cumulative value of 409 (95% CI 86-733) after receiving position intervention for 7 days (**Figure 2A**). This was accompanied by significantly increased ROX index (**Figure 2B**) and decreased Borg scale (**Figure 2C**) since day 3 with a cumulative value of 26 (95% CI 9-43) and -9 (95% CI -15 to -3), respectively. All these findings are suggestive early position intervention effectively improved oxygenation and reduced dyspnea as compared to standard care.

Chest imaging showed that the CT score for lung lesion was significantly reduced in both groups (**Figure 3A**), with position intervention group: 9.9 ± 1.9, after vs. 17.2 ± 5.6, before ($P < 0.001$) and standard care group: 10.9 ± 2, after vs. 14 ± 6, before ($P < 0.015$). In corroborate with improved oxygenation, early position intervention was also associated with more patients had apparent lung lesion absorption (16/17, 94% vs. 11/35, 31%, $P < 0.001$; **Figure 3B and Table 2**). Representative chest CT images of a patient who underwent prone positioning are presented in **Figure 3C**.

The proportion of patients with high NEWS2 (≥ 7) in the position intervention group was significantly lower on days 7 (11.8% vs. 51.4%, $P$ = 0.009) and 14 (11,8% vs. 21.2%, $P$ = 0.007) than those who were in the standard care group (**Table 2**). There was a trend toward shortened time to clinical improvement in the position intervention group over the standard care

ORIGINAL UNEDITED MANUSCRIPT

group, albeit no statistical significance ($P = 0.138$) had been reached (**Figure 4**).

No significant difference in rate of intubation avoidance, virus shedding, and length of hospital stay was observed between the two groups (**Table 2**). No adverse events occurred during the study.

Results for clinical outcomes were unaltered between the two groups when intention-to-treat analysis was performed (**Supplementary Figure E2 and Table E4-5**).

**Discussion**

This was the first study to evaluate the efficacy and safety for early use of prone or lateral positioning on awake severe COVID-19 patients receiving non-mechanical ventilation. Our findings indicate that position intervention significantly improved oxygenation along with relieved subjective dyspnea and facilitated absorption of lung lesions shown in chest images. Further, the position intervention reduced proportion of patients with high NEWS2 on days 7 and 14, and there was a trend toward reduced time to clinical improvement. The position intervention is not correlated with the virus shedding, which may have influence on the length of hospital stay. So the time to virus shedding and length of hospital stay showed no difference between two groups (P＞0.05). The prone or lateral positioning was safely performed and well tolerated by our patients.

ARDS is the main reason of COVID-19 patients for ICU admission with an incidence of 6.2% in Chinese population (3) and 9% in Lombardy, Italy (4), during the pandemic period with a mortality 51% and 26%, respectively. Our

ORIGINAL UNEDITED MANUSCRIPT

chest CT images of severe COVID-19 patients predominantly showed exudation and consolidation near the dorsal distribution in the peripheral lung (20). These imaging findings are in accord with post-mortem pathological appearance of the lungs from deceased COVID-19 patients. In these patients, the alveolar septa were congested and edematous along with serous fibrin exudate and hyaline membrane seen in the alveolar cavity, typical signs indicative of ARDS (14). The admission features of severe COVID-19 patients were similar to those who were managed in the ICU settings in China. These patients were order and had longer time from onset to hospital admission, more comorbid hypertension, diabetes, cardiovascular and chronic lung diseases, higher platelets and D-dimer, and lower lymphocytes compared to non-severe, non-ICU patients (**Supplementary Tables E1-3**).

Many severe COVID-19 patients will rapidly progress to refractory hypoxia, severe ARDS, an irreversible stage in which invasive mechanical ventilation is inevitably. Therefore, we believe early intervention may protect against severe COVID-19 patients transitioning to the critical stage. Prone positioning during invasive mechanical ventilation for ARDS has been extensively studied and improved oxygenation and lung recruitment and may decease mortality (23). Nevertheless, early prone positioning is recommended for sedated patients with a threshold $PaO_2/FiO_2 < 150$ mmHg, the feasibility and timing of its application has never been appraised in awake severe patients with COVID-19. In our study, we had 59% (10/17) of patients with admission $PaO_2/FiO_2 <$ 150 mmHg in the position intervention group who had faster oxygenation improvement compared with those the standard care group (63%, 22/35). These findings imply that early position intervention in these patients may

ORIGINAL UNEDITED MANUSCRIPT

protect against them to progress to severe ARDS and avoid intubation. A recent meta-analysis has shown a longer prone positioning time (12 hours or more) was associated with lower mortality in patients with moderate to severe ARDS (9). However, since it was difficult for our patients to hold the unusual position for 12 hours, a 4-hour positioning procedure was adopted. It appeared that this procedure was well tolerated by the 17 out of 20 patients, even in patients with comorbid cardiovascular and lung diseases. The reason for 3 dropped out patients were mainly due to subjective incompliance rather than actual intolerance or disease severity. Patient education and encouragement may further improve tolerability and reduce nursing burdens especially for the scarce ICU resources during this global pandemic (24).

In this study, we used the oxygen saturation index ($SpO_2/FiO_2$) to evaluate the oxygenation level of the patients. $SpO_2/FiO_2$ is reliable non-invasive surrogate maker for $PaO_2/FiO_2$ (25, 26) and is more practical for continuous oxygenation monitoring in awake COVID-19 patients. ROX index was calculated by $SpO_2/FiO_2$ to RR and was reported to have an additive effect on the accuracy for discriminating between succeeded and failed patients who received high-flow nasal cannula oxygen therapy (27, 28). Here we used the ROX index to help assessing the clinical improvement or deterioration. Borg scale was recommended measures for breathlessness from 0 to 10 score (16). It was easy to use and added a layer of reliability for the improved oxygenation determined by $SpO_2/FiO_2$ and ROX index in the position intervention group. The improvement in oxygenation parameters were also simultaneously associated with accelerated lung lesion absorption. Previous studies have investigated the mechanisms of position intervention in

ORIGINAL UNEDITED MANUSCRIPT

improving oxygenation: gravitational influence results in increased perfusion to the well-ventilated lung, which could enhance the ventilation-perfusion mismatch (29); prone positioning promotes the re-expansion of the collapsed alveoli and improves oxygenation, which is in turn conducive to secretions elimination (30). These mechanisms may also explain the effect of prone position on the clinical improvement of patients with severe COVID-19 in our study.

Patients with early prone position intervention had a lower NEWS2 on days 7 and 14 and appeared to have as shortened time to clinical improvement. All these indicated that position intervention might reduce the risk of COVID-19 progression. Although no significant difference was detected in avoidance of mechanical ventilation between the two groups due to a small sample size, none of the patients in the position intervention group required invasive mechanical ventilation. These results were similar to a previous study, in which it was found that early prone positioning in patients of moderate ARDS with an initial $SpO_2$ > 95% on non-invasive ventilation may benefit and avoid the need for invasive ventilation (23).

Interpreting the results of this study is limited by the small sample size, lack of randomization, drop-out patients in the intervention group. Nevertheless, the combination of oxygenation parameters ($SpO_2$/$FiO_2$, ROX, and Borg scale) had been consistently observed to be improved by the position intervention, and this was also strongly supported by improved imaging of lung lesion abortion. To minimize potential bias for not being randomized, we set up the same protocols for daily management and the

ORIGINAL UNEDITED MANUSCRIPT

same criteria for imaging and discharge. Moreover, the intention-to-treat analysis did not change the overall results obtained.

In conclusion, this was the first study demonstrated that early prone or lateral positioning could improve clinical manifestation and radiological features in non-invasively ventilated severe COVID-19 patients. Because position intervention is simple, inexpensive, and safe, it may be adopted as a regular procedure for the early management of severe COVID-19 patients without invasive ventilation. However, base on the study limitations (small number of patients, nonrandomized trial) , a randomized controlled trial could be recommended to confirm this hypothesis in the future.

**Author Contributions:** D.L., F.L., J.L., and W.L. contributed to the conception of the study. Z.N., K.W., T.F., J.D., Z.Q., J.L., S.X., Y.D., and D.L. participated to daily patient management. Z.N., K.W., T.W, Y.N., Y.W., B.W., and D.L. contributed to data collection. P.Z. performed statistical analysis. Z.N., K.W., T.W., P.Z., W.H., and D.L. contributed to data analysis and interpretation. W.C. collected data from literature reporting clinical features of COVID-19 patients. Z.N., K.W., T.W., W.H., and D.L. drafted the manuscript. G.W., Z.L., W.L., J.L., and F.L. revised the manuscript for important intellectual content. All authors approved the final version of the manuscript.

**Acknowledgements:**

This work was supported by the National Key Development Plan for Precision Medicine Research (2017YFC0910004), Sichuan Science and Technology Project (2020YFS0005 and 2020YFS0009), and the 1.3.5 Project for

ORIGINAL UNEDITED MANUSCRIPT

Disciplines of Excellence–Clinical Research Incubation Project, West China Hospital, Sichuan University (No. ZYJC18001). We also would like to thank two radiologists Drs. Zixing Huang and Qi Leng who helped with the analysis and interpretation of the imaging data.

**Conflict of interest**

The authors declare no competing interests.

**Figure 1**

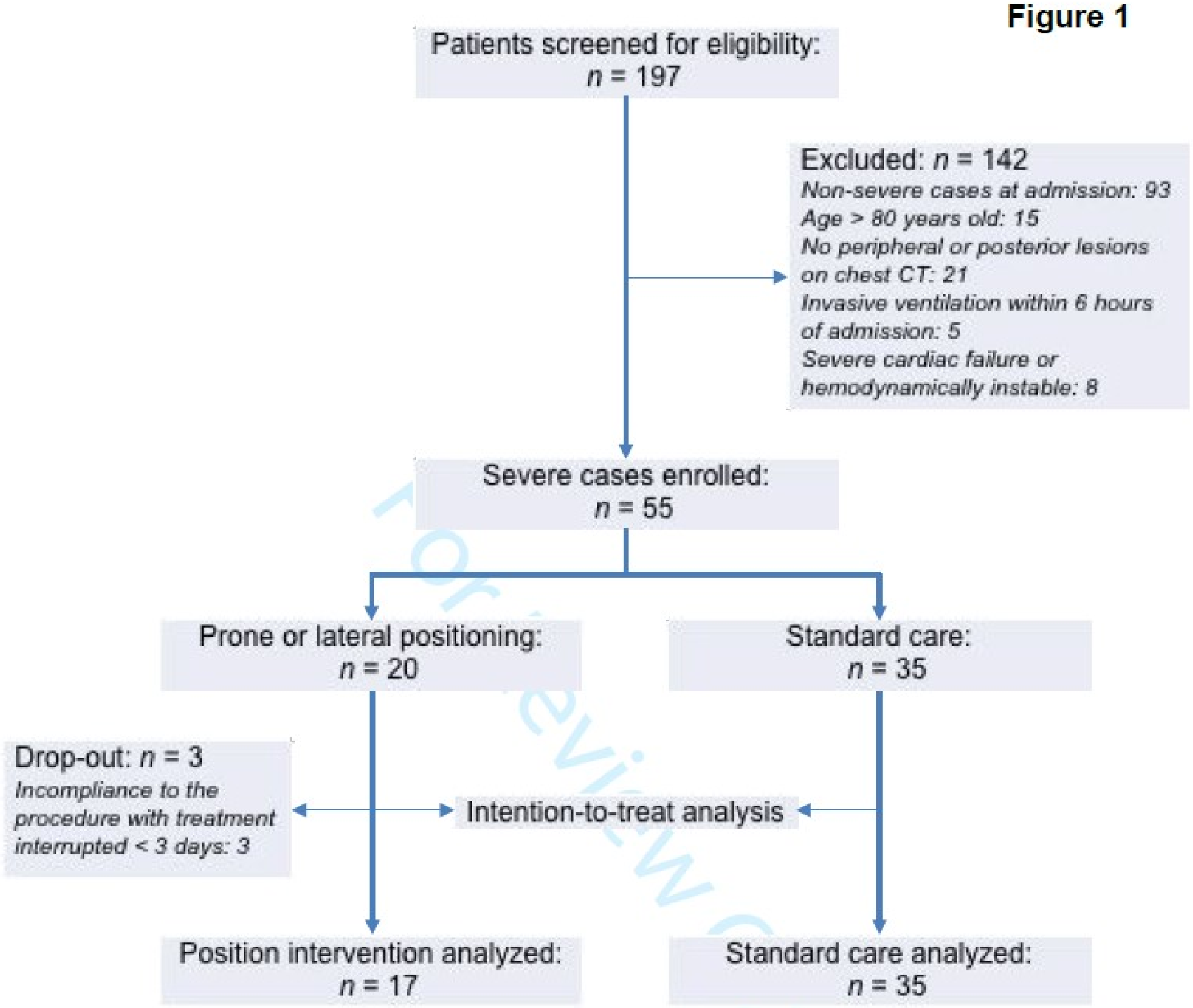

**Figure 1.** Flow chart of patient inclusion.

ORIGINAL UNEDITED MANUSCRIPT

Figure 2

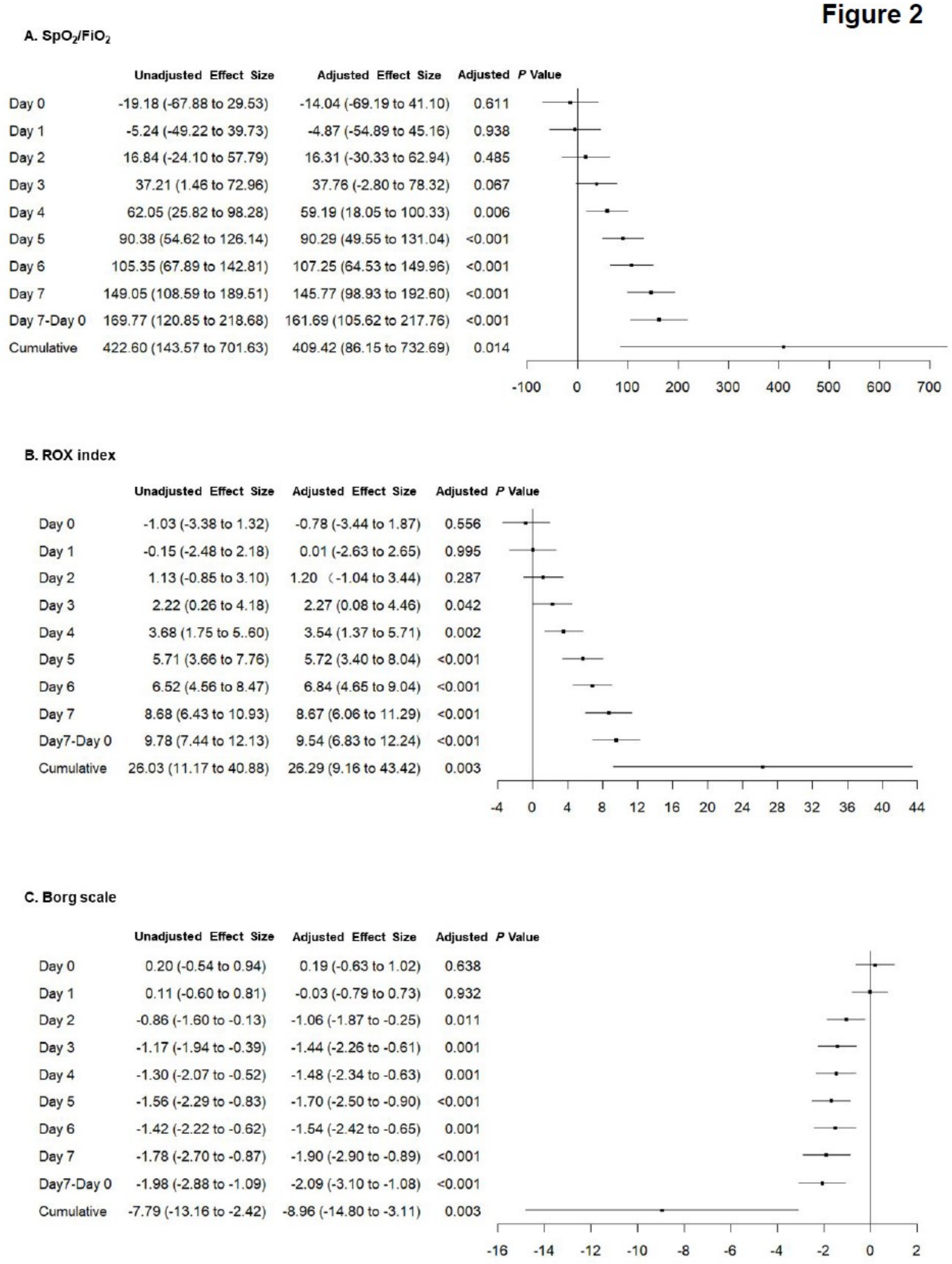

**Figure 2.** Forest plots showing between-group mean difference of position intervention vs. standard care. (A) Pulse oximetry to fraction of inspired oxygen ($SpO_2/FiO_2$). (B) Respiratory rate-Oxygenation (ROX) index. (C) Borg scale. Data are expressed as mean (95% CI).

**Figure 3**

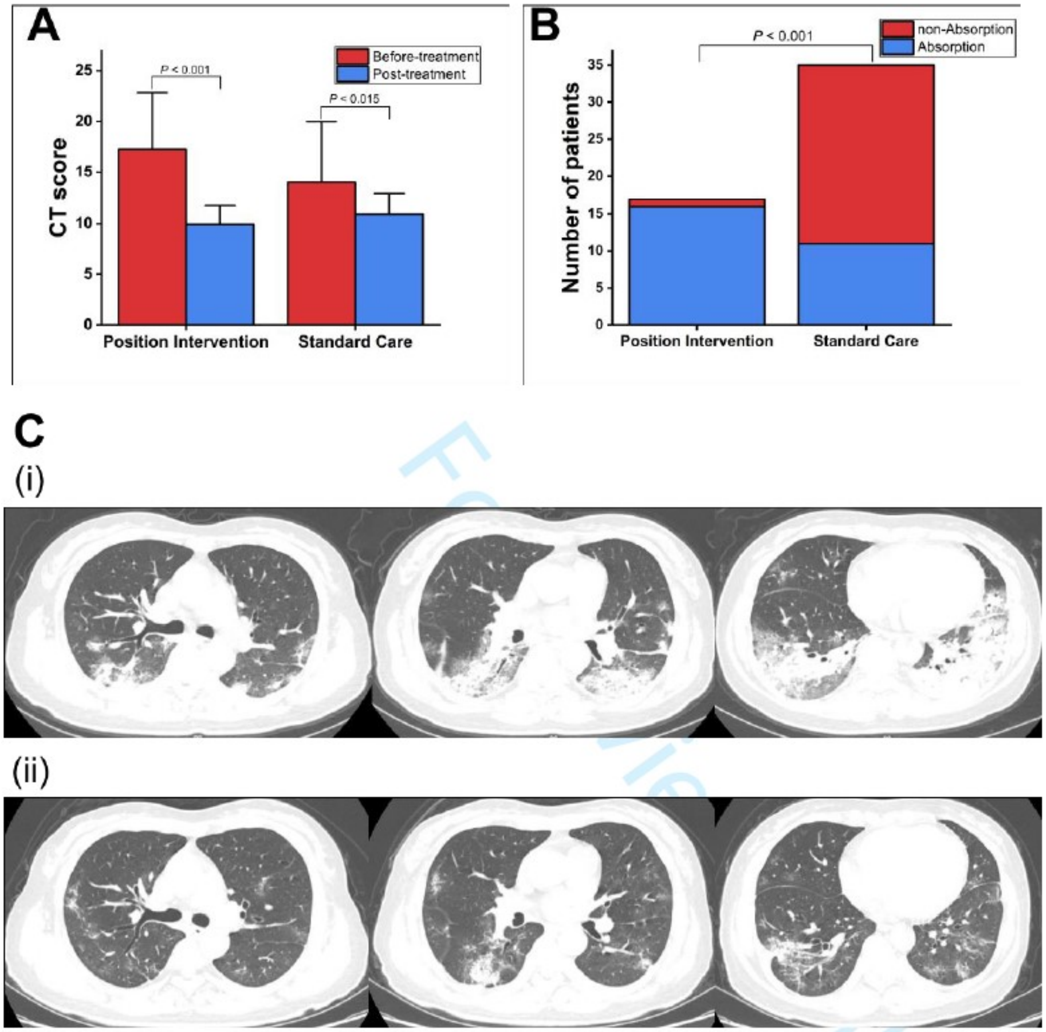

**Figure 3.** Chest CT score and changes before and after treatment. (A) Chest CT score. (B) Number of patients with lung lesion absorption > 30%. (C) Chest CT images of a 52-year-old patient with COVID-19 A: (i) Chest CT images obtained on February 11th, 2020 show predominantly bilateral posterior consolidation. (ii) Chest CT images obtained on February 20th, 2020 show the absorption of consolidation after prone position treatment from February 13th to February 20th, 2020.

Figure 4

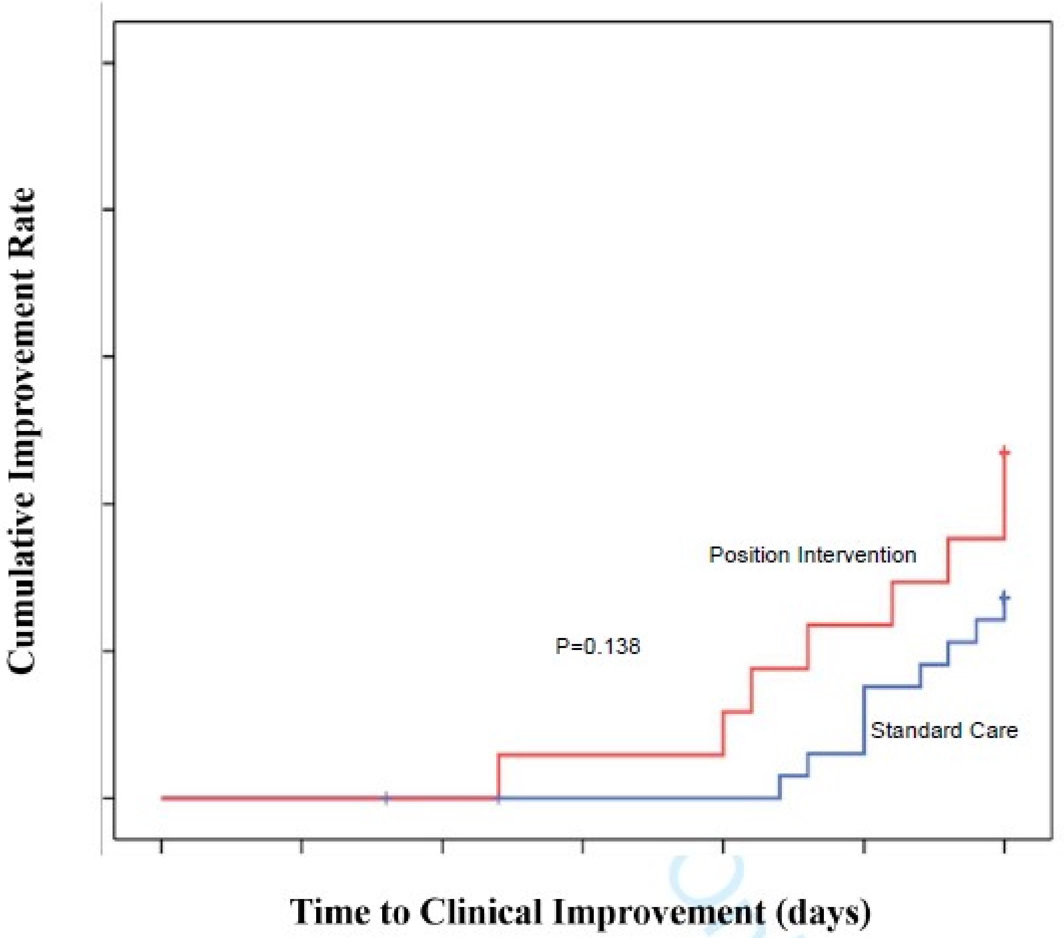

**Figure 4.** Time to clinical improvement.

ORIGINAL UNEDITED MANUSCRIPT

**Table 1.** Baseline Characteristics of Patients at Enrolment

| Characteristics | Total included (*n = 52*) | Position intervention (*n = 17*) | Standard care (*n = 35*) | *P* value |
|---|---|---|---|---|
| Demographics | | | | |
| Age, yr, mean (SD) | 62 ± 12 | 60 ± 12 | 64 ± 12 | 0.265 |
| Age > 60 yr, n (%) | 35 (67.3) | 10 (58.8) | 25 (71.4) | 0.363 |
| Gender, male, n (%) | 32 (61.5) | 11 (64.7) | 21 (60.0) | 0.744 |
| Overall comorbidity, n (%) | 33 (63.5) | 10 (58.8) | 23 (65.7) | 0.628 |
| Hypertension, n (%) | 17 (32.7) | 7 (41.2) | 10 (28.6) | 0.363 |
| Diabetes, n (%) | 11 (21.2) | 4 (23.5) | 7 (20.0) | 1.000 |
| Coronary heart disease, n (%) | 7 (13.5) | 3 (17.6) | 4 (11.4) | 0.855 |
| COPD, n (%) | 6 (11.5) | 3 (17.6) | 3 (8.6) | 0.618 |
| Time to admission, d, median (IQR) | 10 (7-14) | 10 (7-13) | 10 (7-15) | 0.448 |
| Oxygenation status and severity | | | | |
| Respiratory rate, breaths/min, | 25 ± 5 | 23 ± 4 | 26 ± 5 | 0.057 |
| mean (SD) | 27 (51.9) | 11 (64.7) | 16 (45.7) | 0.199 |
| < 25 — n (%) | 25 (48.1) | 6 (35.3) | 19 (54.3) | |
| ≥ 25 — n (%) | | | | |
| $PaO_2/FiO_2$, mmHg, mean (SD) | 133 ± 58 | 142 ± 54 | 128 ± 60 | 0.426 |
| ≥ 200 to < 300 — n (%) | 9 (17.3) | 3 (17.6) | 6 (17.1) | 1.000 |
| ≥ 100 to < 200 — n (%) | 27 (52.0) | 9 (53.0) | 18 (51.4) | |
| < 100 — n (%) | 16 (30.8) | 5 (29.4) | 11 (31.4) | |
| PSI, mean (SD) | 99 ± 20 | 101 ± 25 | 98 ± 17 | 0.582 |
| ≤ 90 — n (%) | 15 (28.8) | 6 (35.3) | 9 (25.7) | 0.501 |
| 91-130 — n (%) | 35 (67.3) | 9 (53.0) | 26 (74.3) | |
| > 130 — n (%) | 2 (3.8) | 2 (11.8) | 0 | |
| NEWS2, median (IQR) | 8 (7-9) | 9 (8-10) | 8 (7-9) | 0.317 |
| 1-4 — n (%) | 0 | 0 | 0 | 0.161 |
| 5-6 — n (%) | 6 (11.5) | 0 | 6 (17.1) | |
| ≥ 7 — n (%) | 46 (88.5) | 17 (100.0) | 29 (82.9) | |

ORIGINAL UNEDITED MANUSCRIPT

| | | | | |
|---|---|---|---|---|
| Laboratory indices | | | | |
| Hemoglobin, g/L, median (IQR) | 127 (116-138) | 130 (120-137) | 127 (110-139) | 0.232 |
| < 120 — n (%) | 16 (30.8) | 4 (23.5) | 12 (34.3) | 0.43 |
| ≥ 120 — n (%) | 36 (69.2) | 13 (76.5) | 23 (65.7) | |
| WBC count, ×$10^9$/L, median (IQR) | 7.08 (5.75-10.31) | 7.18 (5.65-9.61) | 7.04 (5.65-11.23) | 0.728 |
| 4–10 — n (%) | 37 (71.2) | 13 (76.5) | 24 (68.6) | 0.554 |
| < 4 — n (%) | 2 (3.8) | 1 (5.9) | 1 (2.9) | |
| > 10 — n (%) | 13 (25.0) | 3 (17.6) | 10 (28.6) | |
| Lymphocyte count, ×$10^9$/L, median (IQR) | 0.67 (0.49-0.93) | 0.62 (0.51-0.79) | 0.68 (0.48-0.95) | 0.903 |
| ≥ 1.0 — n (%) | 9 (17.3) | 2 (11.8) | 7 (20.0) | 0.730 |
| < 1.0 — n (%) | 43 (82.7) | 15 (88.2) | 28 (80.0) | |
| Platelet count, ×$10^9$/L, median (IQR) | 228 (172-290) | 191 (165-268) | 251 (167-313) | 0.814 |
| ≥100 — n (%) | 49 (94.2) | 15 (88.2) | 34 (97.1) | 0.246 |
| <100 — n (%) | 3 (5.8) | 2 (11.8) | 1 (2.9) | |
| ALT, U/L, median (IQR) | 36 (23-61) | 28.5 (22.25-43) | 45 (23-65.75) | 0.728 |
| ≤ 40 — n (%) | 29 (55.8) | 12 (70.6) | 17 (48.6) | 0.134 |
| > 40 — n (%) | 23 (44.2) | 5 (29.4) | 18 (51.4) | |
| AST, U/L, median (IQR) | 33 (26-46) | 30 (23-41) | 37 (30-48) | 0.397 |
| ≤ 50 — n (%) | 43 (82.7) | 15 (88.2) | 28 (80.0) | 0.730 |
| > 50 — n (%) | 9 (17.3) | 2 (11.8) | 7 (20.0) | |
| Creatine, μmol/ L, median (IQR) | 64 (50-75) | 67(52-85) | 63 (48-71) | 0.336 |
| ≤ 133 — n (%) | 49 (94.2) | 16 (94.1) | 33 (94.3) | 1.000 |
| > 133 — n (%) | 3 (5.8) | 1 (5.9) | 2 (5.7) | |
| LDH, U/L, median (IQR) | 375 (306-479) | 412 (310-478) | 366 (302-522) | 0.250 |
| ≤ 245 — n (%) | 4 (7.7) | 1 (5.9) | 3 (8.6) | 1.000 |
| > 245 — n (%) | 48 (92.3) | 16 (94.1) | 32 (91.4) | |
| CK-MB, ng/L, median (IQR) | 1.18 (0.79-2.01) | 0.91 (0.66-1.98) | 1.35 (0.94-2.13) | 0.499 |
| ≤ 5 — n (%) | 50 (96.2) | 15 (88.2) | 35 (100.0) | 0.103 |
| > 5 — n (%) | 2 (3.8) | 2 (11.8) | 0 | |
| BNP, pg/L, median (IQR) | 323 (204-589) | 303 (188-457) | 358 (204-607) | 0.693 |

ORIGINAL UNEDITED MANUSCRIPT

| | | | | |
|---|---|---|---|---|
| ≤ 450 — n (%) | 24 (46.2) | 3 (17.6) | 21 (60.0) | 0.004 |
| > 450 — n (%) | 28 (53.8) | 14 (82.4) | 14 (40.0) | |
| CRP, mg/L, median (IQR) | 73 (46-120) | 73 (41-117) | 73 (47-121) | 0.976 |
| ≤ 5 — n (%) | 2 (3.8) | 1 (5.9) | 1 (2.9) | 1.000 |
| > 5 — n (%) | 50 (96.2) | 16 (94.1) | 34 (97.1) | |
| PCT, ng/mL, median (IQR) | 0.09 (0.05-0.23) | 0.09 (0.07-0.23) | 0.10 (0.05-0.24) | 0.435 |
| < 0.1 — n (%) | 28 (53.8) | 11 (64.7) | 17 (48.6) | 0.274 |
| ≥ 0.1 — n (%) | 24 (46.2) | 6 (35.3) | 18 (51.4) | |
| TNI, ng/L, median (IQR) | 0.01 (0.01-0.01) | 0.01 (0.01-0.02) | 0.01 (0.01-0.01) | 0.682 |
| ≤ 0.04 — n (%) | 51 (98.1) | 17 (100.0) | 34 (97.1) | 1.000 |
| > 0.04 — n (%) | 1 (1.9) | 0 | 1 (2.9) | |
| PT, second, median (IQR) | 12.3 (11.7-12.8) | 12.2 (11.65-12.85) | 12.3 (11.7-12.8) | 0.908 |
| 9-13 — n (%) | 45 (86.5) | 14 (82.4) | 31 (88.6) | 0.855 |
| ≥ 13 — n (%) | 7 (13.5) | 3 (17.6) | 4 (11.4) | |
| D-dimer, mg/L, median (IQR) | 1.9 (1.18-4.18) | 1.46 (0.86-3.38) | 2.35 (1.21-5.05) | 0.214 |
| ≤ 0.55 — n (%) | 3 (5.8) | 1 (5.9) | 2 (5.7) | 1.000 |
| > 0.55 — n (%) | 49 (94.2) | 16 (94.1) | 33 (94.3) | |

*Definition of abbreviations:* COPD=Chronic obstructive pulmonary disease; $PaO_2/FiO_2$= partial pressure of arterial oxygen to fraction of inspired oxygen ratio; PSI=Pneumonia Severity Index; NEWS2=National Early Warning Score 2; SBP=systolic blood pressure; DBP=diastolic blood pressure; WBC=white blood cell; ALT=alanine transaminase; AST=aspartate aminotransferase; LDH=lactate dehydrogenase; CK-MB=creatine kinase myocardial band; BNP= B-type natriuretic peptide; CRP=C-reactive protein; PCT=procalcitonin; TNI=troponin I; PT=prothrombin time.

ORIGINAL UNEDITED MANUSCRIPT

**Table 2.** Treatment of the Patients after Enrolment

| **Characteristics** | Total (*n=52*) | Position intervention (*n = 17*) | Standard care (*n = 35*) | *P* value |
|---|---|---|---|---|
| Respiratory support during study, n (%) | | | | 0.916 |
| Nasal cannula and mask | 37 (71.2) | 13 (76.5) | 24 (68.6) | |
| High flow ventilation | 11 (21.2) | 3 (17.6) | 8 (22.9) | |
| Non-invasive mechanical ventilation | 3 (5.8) | 1 (5.9) | 2 (5.7) | |
| Invasive mechanical ventilation | 1 (1.9) | 0 (0.0) | 1 (2.9) | |
| Treatment | | | | |
| Antivirals, n (%) | 50 (100) | 17 (100) | 35 (100) | - |
| Antibiotics, n (%) | 44 (84.6) | 13 (76.5) | 31 (88.6) | 0.469 |
| Anticoagulants, n (%) | 10 (19.2) | 2 (11.8) | 8 (22.9) | 0.564 |
| Glucocorticoids, n (%) | 34 (65.4) | 12 (70.6) | 22 (62.9) | 0.583 |
| Glucocorticoid duration, d,median (IQR) | 3 (0-6) | 3 (0-6) | 3 (0-6) | 0.520 |
| CT absorption > 30%, n (%) | | | | <0.001 |
| No | 25 (48.1) | 1 (5.9) | 24 (68.6) | |
| Yes | 27 (51.9) | 16 (94.1) | 11 (31.4) | |
| NEWS2 (d 7), median (IQR) | 6 (5-8) | 5 (5-5) | 6 (5-8) | 0.002 |
| 1-4 | 10 (19.2) | 6 (35.3) | 4 (11.4) | 0.009 |
| 5-6 | 22 (42.3) | 9 (52.9) | 13 (37.1) | |
| ≥ 7 | 20 (38.5) | 2 (11.8) | 18 (51.4) | |
| NEWS2 (d 14), median (IQR) | 4 (3-6) | 3 (2-3) | 5 (4-7) | <0.001 |
| 1-4 | 31 (62.0) | 15 (88.2 ) | 16 (48.5) | 0.007 |
| 5-6 | 10 (20.0) | 0 (0) | 10 (30.3) | |
| ≥ 7 | 9 (18.0) | 2 (11.8) | 7 (21.2) | |
| Rate of intubation avoidance, n (%) | 51 (98) | 17 (100) | 34 (97.1) | 1.000 |
| Time to clinical improvement, d, median (IQR) | 33 (27-41) | 31 (25-40) | 35 (28-42) | 0.234 |
| Time to virus shedding, d, median (IQR) | 26 (19-33) | 30 (20-36) | 25 (18-31) | 0.315 |
| Length of hospital stay, d, median (IQR) | 35 (28-43) | 35 (27-52) | 35 (28-42) | 0.914 |
| Adverse events, n (%) | 0 (0) | 0 (0) | 0 (0) | |

*Definition of abbreviations:* NEWS2=National Early Warning Score 2.

ORIGINAL UNEDITED MANUSCRIPT
