# Efficacy of early prone or lateral positioning in patients with severe COVID-19: a single-center prospective cohort

**DOI:** 10.1093/pcmedi/pbaa034

**Published:** 2020-09-28

**Authors:** Zhong Ni, Kaige Wang, Ting Wang, Yuenan Ni, Wei Huang, Ping Zhu, Tao Fan, Ye Wang, Bo Wang, Jun Deng, Zhicheng Qian, Jiasheng Liu, Wenhao Cai, Shanling Xu, Yu Du, Gang Wang, Zongan Liang, Weimin Li, Jianfei Luo, Fengming Luo, Dan Liu

**Affiliations:** Department of Respiratory and Critical Care Medicine, Clinical Research Center for Respiratory Disease, West China Hospital, Sichuan University, Chengdu 610041, China; Department of Respiratory and Critical Care Medicine, Clinical Research Center for Respiratory Disease, West China Hospital, Sichuan University, Chengdu 610041, China; Department of Respiratory and Critical Care Medicine, Clinical Research Center for Respiratory Disease, West China Hospital, Sichuan University, Chengdu 610041, China; Department of Respiratory and Critical Care Medicine, Clinical Research Center for Respiratory Disease, West China Hospital, Sichuan University, Chengdu 610041, China; Department of Integrated Traditional Chinese and Western Medicine, West China Hospital, Sichuan University, Chengdu 610041, China; Department of Integrated Traditional Chinese and Western Medicine, West China Hospital, Sichuan University, Chengdu 610041, China; Department of Integrated Traditional Chinese and Western Medicine, West China Hospital, Sichuan University, Chengdu 610041, China; Department of Respiratory and Critical Care Medicine, Clinical Research Center for Respiratory Disease, West China Hospital, Sichuan University, Chengdu 610041, China; Department of Respiratory and Critical Care Medicine, Clinical Research Center for Respiratory Disease, West China Hospital, Sichuan University, Chengdu 610041, China; Department of Respiratory and Critical Care Medicine, The Affiliated Hospital of Southwest Medical University, Luzhou 646000, China; Intensive Care Unit, the Affiliated Hospital of North Sichuan Medical College, Nanchong 637000, China; Department of Gastrointestinal Surgery, Renmin Hospital of Wuhan University, Wuhan 430060, China; Department of Integrated Traditional Chinese and Western Medicine, West China Hospital, Sichuan University, Chengdu 610041, China; Critical Care Medicine Department, Sichuan Cancer Hospital, Affiliated Cancer Hospital to University of Electronic Science and Technology of China, Chengdu 610041, China; Department of Emergency and Critical Care Medicine, West China School of Public Health, West China Fourth Hospital, Sichuan University, Chengdu 610041, China; Department of Respiratory and Critical Care Medicine, Clinical Research Center for Respiratory Disease, West China Hospital, Sichuan University, Chengdu 610041, China; Department of Respiratory and Critical Care Medicine, Clinical Research Center for Respiratory Disease, West China Hospital, Sichuan University, Chengdu 610041, China; Department of Respiratory and Critical Care Medicine, Clinical Research Center for Respiratory Disease, West China Hospital, Sichuan University, Chengdu 610041, China; Department of Gastrointestinal Surgery, Renmin Hospital of Wuhan University, Wuhan 430060, China; Department of Respiratory and Critical Care Medicine, Clinical Research Center for Respiratory Disease, West China Hospital, Sichuan University, Chengdu 610041, China; Department of Respiratory and Critical Care Medicine, Clinical Research Center for Respiratory Disease, West China Hospital, Sichuan University, Chengdu 610041, China

**Keywords:** COVID-19, early position intervention, oxygenation, ROX index, Borg scale

## Abstract

**Background:**

Position intervention has been shown to improve oxygenation, but its role in non-invasively ventilated patients with severe COVID-19 has not been assessed. The objective of this study was to investigate the efficacy of early position intervention on non-invasively ventilated patients with severe COVID-19.

**Methods:**

This was a single-center, prospective observational study in consecutive patients with severe COVID-19 managed in a provisional ICU at Renmin Hospital of Wuhan University from 31 January to 15 February 2020. Patients with chest CT showing exudation or consolidation in bilateral peripheral and posterior parts of the lungs were included. Early position intervention (prone or lateral) was commenced for > 4 hours daily for 10 days in these patients, while others received standard care.

**Results:**

The baseline parameters were comparable between the position intervention group (*n* = 17) and the standard care group (*n* = 35). Position intervention was well-tolerated and increased cumulative adjusted mean difference of SpO_2_/FiO_2_ (409, 95% CI 86 to 733) and ROX index (26, 95% CI 9 to 43) with decreased Borg scale (−9, 95% CI −15 to −3) during the first 7 days. It also facilitated absorption of lung lesions and reduced the proportion of patients with high National Early Warning Score 2 (≥ 7) on days 7 and 14, with a trend toward faster clinical improvement. Virus shedding and length of hospital stay were comparable between the two groups.

**Conclusions:**

This study provides the first evidence for improved oxygenation and lung lesion absorption using early position intervention in non-invasively ventilated patients with severe COVID-19, and warrants further randomized trials.

## Introduction

In December 2019 in Wuhan, Central China, there was an outbreak of coronavirus disease 2019 (COVID-19), characterized by atypical pneumonia.^1^ This has caused a global pandemic with increasing incidence, mortality, medical resource consumption, and social-economic burdens. The full spectrum of COVID-19 ranges from mild, self-limiting respiratory tract illness to severe progressive pneumonia, predominately manifesting as acute respiratory distress syndrome (ARDS) requiring admission to the intensive care unit (ICU).^2^ The rate of invasive mechanical ventilation and mortality of patients with severe COVID-19 remains high, partially attributed to delayed admission and intervention during the outbreak.^3,4^ Despite several endeavors with randomized, placebo-controlled trials of lopinavir-ritonavir^5^ and compassionate use of remdesivir,^6^ chloroquine,^7^ and convalescent plasma,^8^ to date there is no approved pharmacological treatment with definite efficacy for this devastating disease.

Prone positioning has been shown to improve oxygenation and reduce complications and mortality in patients with moderate-to-severe ARDS with invasive mechanical ventilation.^9,10^ Likewise, lateral positioning has been associated with improved pulmonary gas exchange and drainage of secretions in critically ill patients, albeit the efficacy was inconclusive.^11^ The latest guidance issued by the National Health Commission (NHC) of China and the World Health Organization (WHO) for management of COVID-19 recommended only that prone positioning could be applied in critically ill patients who were under invasive mechanical ventilation. A recent preliminary study of 12 patients critically ill with COVID-19 showed that position intervention was associated with improved oxygenation and lung recruitability.^12^ However, the effects of position intervention have not been assessed in awake patients with severe COVID-19 without intubation at admission.

The objective of this study was to investigate the impact of early prone or lateral positioning on oxygenation improvement, lung lesion absorption, and other clinical outcomes in awake patients with severe COVID-19.

## Methods

### Study design and patients

This prospective, observational cohort study, was designed, conducted, and reported according to Strengthening the Reporting of Observational Studies in Epidemiology (STROBE) guidelines.^13^ Consecutive patients with COVID-19 admitted to provisional ICUs in Renmin Hospital of Wuhan University (between 31 January and 15 February 2020) were scrutinized for inclusion. This study sought to compare the clinical outcomes between patients who received early position intervention and those who had standard care only. The study protocol was reviewed and approved by the Institutional Review Board of Renmin Hospital of Wuhan University (No. WDRY2020-K068). On a separate note, studies in China comparing patients in ICU with non-ICU or patients with severe disease with non-severe were manually searched (by W.C. to 20 April 2020) to understand the baseline characteristics of these patients.

Diagnosis of COVID-19 was defined as the presence of severe acute respiratory syndrome coronavirus 2 before admission, determined by a reverse transcription-polymerase chain reaction method as per NHC of China^14^ and WHO^15^ standards. Patients were included in this study if they met the following criteria: classified as severe category of COVID-19, manifesting as dyspnea with respiratory rate (RR) ≥ 30 breaths/min, pulse oxygen saturation ≤ 93% at rest, or partial pressure of arterial oxygen (PaO_2_) to fraction of inspired oxygen (FiO_2_) ratio ≤ 300 mmHg^14^; with chest computerized tomographic (CT) images showing exudation or consolidation mainly in the bilateral peripheral and posterior parts of the lungs.

Patients were excluded if they were aged < 18 or > 80 years old; were pregnant; were critically ill (invasive mechanical ventilation, severe cardiac failure, or hemodynamically unstable)^14^; contraindicated to the prone or lateral position (i.e. a history of the vertebral disease); or were unable to cooperate.

Three attending physicians independently analyzed eligibility of patients for recruitment (Z.L., J.L., and T.W.). If there was a disagreement, the final decision was made by the medical team leader (D.L.).

### Interventions and measurements

All patients received standard care according to the interim guidance version 4 issued by the NHC of China.^14^ According to whether or not they received position intervention, the patients were divided into two groups:

1. Standard care group: standard care comprised supplemental oxygen and ventilation, antivirals (ribavirin or arbidor), antibiotics, anticoagulants, and glucocorticoids, as necessary, based on the clinical condition of the patients. In this group, no position intervention was introduced.
2. Position intervention group: prone positioning was superimposed on the standard care at the doctors’ discretion without pre-defined selection criteria. Where prone positioning was not tolerated by a patient, lateral positioning was implemented as an alternative. Each patient was placed in position for at least 4 hours per day for 10 days. The exact position placement was performed and managed by an experienced respiratory therapist and a physician, and all patients were evaluated every morning.

Vital signs, oxygen saturation measured by pulse oximetry (SpO_2_), FiO_2_, and Borg scale (a 0–10 category scale verbal description for severity of dyspnea)^16^ were recorded between 9 and 11 am daily for at least 7 days. To determine illness severity, the Pneumonia Severity Index (PSI)^17^ was estimated within 24 hours of admission. The risk of clinical deterioration were assessed with three levels of National Early Warning Score 2 (NEWS2): Low score, an aggregate of 1–4 (or a score of 3 in any one parameter); Medium score, an aggregate of 5 or 6, indicative of potential severe acute clinical deterioration and the need for an urgent clinical response; High score, an aggregate score of ≥ 7.^18^ NEWS2 was assessed at enrollment, on days 7 and 14 thereafter or before decreased.

In addition to the chest CT scan prior to admission, all patients in this study underwent follow-up CT examinations on day 10 after enrollment. The imaging results were interpreted by two attending radiologists who were unaware of the study design. The principle of discharge was based on relief of symptoms, obvious absorption of inflammation in chest CT, abatement of fever, and viral clearance with throat swabs two consecutive times more than 24 hours apart.

### Data collection

The data collection process followed quality assurance and standard operating procedures developed by senior authors (W.L., J.L., F.L., and D.L.). Two researchers (Z.L. and J.L.) independently started data collection at enrollment from electronic medical records independently using pre-defined *pro forma*. Data quality were checked by a third researcher (T.W.). These data contained epidemiological, virologic, clinical, laboratory, microbiological, radiological characteristics, disease severity indices, respiratory support method, pharmacological treatment, and other management details.

### Outcome and safety measures

The primary outcome was oxygenation improvement, determined by cumulative adjusted mean difference of SpO_2_/FiO_2_ (serving as oxygen saturation index), Respiratory rate-Oxygenation (ROX) index, and Borg scale between position intervention and standard care.

Secondary outcomes were lung lesion absorption, NEWS2, time to clinical improvement, rate of intubation avoidance, death, time to virus shredding, length of hospital stay, and adverse events. The lung lesion absorption was described as changes in chest CT manifestations after treatment as contrast to admission imaging findings. A semi-quantitative scoring system was used to quantitatively estimate the pulmonary involvement of all abnormalities on the basis of the area involved.^19^ Each of the five lung lobes was visually scored from 0 to 5 as per a previous study: 0, no involvement; 1, < 5% involvement; 2, 25% involvement; 3, 26%–49% involvement; 4, 50%–75% involvement; 5, > 75% involvement. The total CT score was the sum of the individual lobar scores and ranged from 0 (no involvement) to 25 (maximum involvement).^20^ The changes were stratified into obvious absorption (absorption proportion > 30%) and stable or deterioration (absorption proportion ≤ 30%, without absorption or lesion enlargement).

The time to clinical improvement was defined as the time from enrollment to an improvement by 2 points on a seven-category ordinal scale or live discharge from the hospital, whichever came first.^21^ Adverse events for position intervention included hemodynamic instability and pressure sores.^9,22^

### Statistical analysis

Continuous variables are presented as mean (SD) or median (IQR), compared by Mann-Whitney *U* test. Categorical variables are expressed as frequency (percentage), compared by Chi-square or Fisher's exact test as appropriate. Logistic regression models were used to generate propensity scores that estimated the odds of receiving position intervention versus standard care, covariates are age, gender, and heart rate. Adjustment for differences in characteristics on admission was performed using inverse probability of treatment weighting (IPTW) models, to assess the effect of position intervention on association of oxygenation improvement parameters demonstrated as forest plots. The time to clinical improvement was assessed when all patients had reached day 30 from enrollment. Data were censored if patients failed to reach clinical improvement at day 30 or decreased before. Survival analysis was done by Kaplan-Meier estimation (Log-rank test). Intention-to-treat analysis was additionally performed to verify our results.

Statistical analysis was performed using the R statistical computing environment (version 3.63). A two-sided *P* value of 0.05 or less was considered to be statistically significant.

## Results

### Characteristics and treatments of included patients

**Figure 1.**
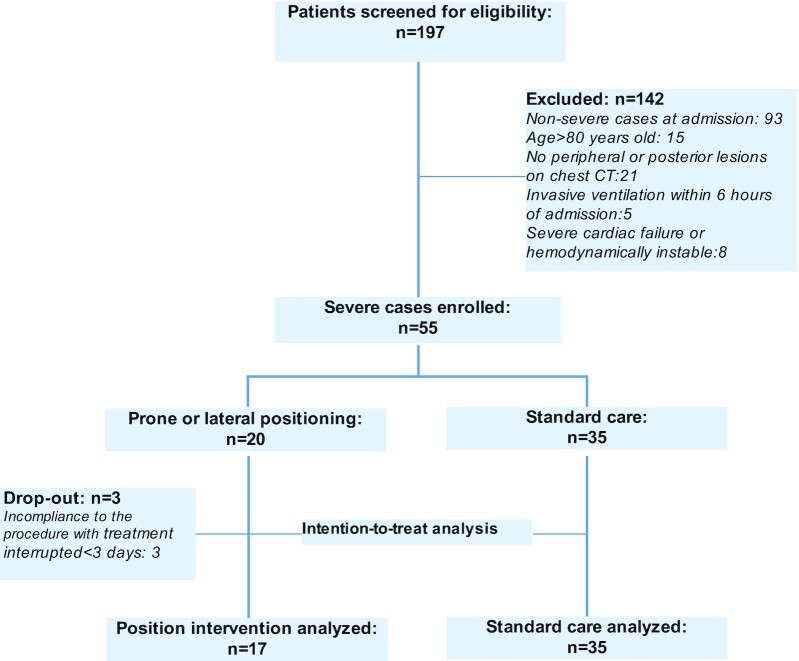
Flow chart of patient inclusion.

The patient selection process is described in Fig. 1. A total of 197 consecutive patients with confirmed diagnosis of COVID-19 was screened for inclusion during the study period. Of these, 142 were considered ineligible for various reasons. The remaining 55 patients with severe disease were included, of whom 20 received position intervention and 35 had standard care only. Three patients in the position intervention group dropped out because of noncompliance with the procedures. Baseline characteristics of position intervention (*n* = 17) vs. standard care (*n* = 35) are compared and summarized in Table 1. The mean age of patients was 62 (SD 12) years, and 32 (62%) were male. Thirty-three patients (64%) had comorbid chronic diseases, including hypertension (33%), diabetes (21%), coronary heart disease (14%), chronic obstructive pulmonary disease (12%), hepatitis B (4%), pulmonary embolism (2%), hypothyroidism (2%), cerebral ischemic stroke (2%), and malignancy (2%). The median time from symptom onset to hospital admission was 10 (IQR 7–14) days. At presentation, the means for respiratory rate, PaO_2_/FiO_2_, PSI were 25 (SD 5), 133 (SD 58), and 99 (SD 20), respectively, and the median for NEWS2 was 8 (IQR 7–9); the medians of lymphocytes (0.67, IQR 0.49–0.93), lactate dehydrogenase (375, IQR 306–479), C-reactive protein (73, IQR 46–120), and D-dimer (1.9, IQR 1.18–4.18) were out of normal range. Chest CT revealed that the predominant pattern of temporal abnormalities was diffuse ground-glass opacity with mainly bilateral and subpleural distributions. These findings are similar to clinical profiles of patients with severe COVID-19 in China, as reported in the literature (Supplementary Tables E1–3).

**Table 1.** Baseline characteristics of patients at enrollment.

Characteristics	Total included (*n* = 52)	Position intervention (*n* = 17)	Standard care (*n* = 35)	*P* value
Demographics				
Age, yr, mean (SD)	62 ± 12	60 ± 12	64 ± 12	0.265
Age > 60 yr, *n* (%)	35 (67.3)	10 (58.8)	25 (71.4)	0.363
Gender, male, *n* (%)	32 (61.5)	11 (64.7)	21 (60.0)	0.744
Overall comorbidity, *n* (%)	33 (63.5)	10 (58.8)	23 (65.7)	0.628
Hypertension, *n* (%)	17 (32.7)	7 (41.2)	10 (28.6)	0.363
Diabetes, *n* (%)	11 (21.2)	4 (23.5)	7 (20.0)	1.000
Coronary heart disease, *n* (%)	7 (13.5)	3 (17.6)	4 (11.4)	0.855
COPD, *n* (%)	6 (11.5)	3 (17.6)	3 (8.6)	0.618
Time to admission, d, median (IQR)	10 (7–14)	10 (7–13)	10 (7–15)	0.448
Oxygenation status and severity				
Respiratory rate, breaths/min, mean (SD)	25 ± 5	23 ± 4	26 ± 5	0.057
< 25—*n* (%)	27 (51.9)	11 (64.7)	16 (45.7)	0.199
≥ 25—*n* (%)	25 (48.1)	6 (35.3)	19 (54.3)	
PaO_2_/FiO_2_, mmHg, mean (SD)	133 ± 58	142 ± 54	128 ± 60	0.426
≥ 200 to < 300—*n* (%)	9 (17.3)	3 (17.6)	6 (17.1)	1.000
≥ 100 to < 200—*n* (%)	27 (52.0)	9 (53.0)	18 (51.4)	
< 100—*n* (%)	16 (30.8)	5 (29.4)	11 (31.4)	
PSI, mean (SD)	99 ± 20	101 ± 25	98 ± 17	0.582
≤ 90—*n* (%)	15 (28.8)	6 (35.3)	9 (25.7)	0.501
91–130—*n* (%)	35 (67.3)	9 (53.0)	26 (74.3)	
> 130—*n* (%)	2 (3.8)	2 (11.8)	0	
NEWS2, median (IQR)	8 (7–9)	9 (8–10)	8 (7–9)	0.317
1–4—*n* (%)	0	0	0	0.161
5–6—*n* (%)	6 (11.5)	0	6 (17.1)	
≥ 7—*n* (%)	46 (88.5)	17 (100.0)	29 (82.9)	
Laboratory indices				
Hemoglobin, g/L, median (IQR)	127 (116–138)	130 (120–137)	127 (110–139)	0.232
< 120—n (%)	16 (30.8)	4 (23.5)	12 (34.3)	0.43
≥ 120—n (%)	36 (69.2)	13 (76.5)	23 (65.7)	
WBC count, × 10^9^/L, median (IQR)	7.08 (5.75–10.31)	7.18 (5.65–9.61)	7.04 (5.65–11.23)	0.728
4–10—n (%)	37 (71.2)	13 (76.5)	24 (68.6)	0.554
< 4—n (%)	2 (3.8)	1 (5.9)	1 (2.9)	
> 10—n (%)	13 (25.0)	3 (17.6)	10 (28.6)	
Lymphocyte count, × 10^9^/L, median (IQR)	0.67 (0.49–0.93)	0.62 (0.51–0.79)	0.68 (0.48–0.95)	0.903
≥ 1.0—*n* (%)	9 (17.3)	2 (11.8)	7 (20.0)	0.730
< 1.0—*n* (%)	43 (82.7)	15 (88.2)	28 (80.0)	
Platelet count, × 10^9^/L, median (IQR)	228 (172–290)	191 (165–268)	251 (167–313)	0.814
≥100—*n* (%)	49 (94.2)	15 (88.2)	34 (97.1)	0.246
<100—*n* (%)	3 (5.8)	2 (11.8)	1 (2.9)	
ALT, U/L, median (IQR)	36 (23–61)	28.5 (22.25–43)	45 (23–65.75)	0.728
≤ 40—*n* (%)	29 (55.8)	12 (70.6)	17 (48.6)	0.134
> 40—*n* (%)	23 (44.2)	5 (29.4)	18 (51.4)	
AST, U/L, median (IQR)	33 (26–46)	30 (23–41)	37 (30–48)	0.397
≤ 50—*n* (%)	43 (82.7)	15 (88.2)	28 (80.0)	0.730
> 50—*n* (%)	9 (17.3)	2 (11.8)	7 (20.0)	
Creatine, μmol/L, median (IQR)	64 (50–75)	67(52–85)	63 (48–71)	0.336
≤ 133—*n* (%)	49 (94.2)	16 (94.1)	33 (94.3)	1.000
> 133—*n* (%)	3 (5.8)	1 (5.9)	2 (5.7)	
LDH, U/L, median (IQR)	375 (306–479)	412 (310–478)	366 (302–522)	0.250
≤ 245—*n* (%)	4 (7.7)	1 (5.9)	3 (8.6)	1.000
> 245—*n* (%)	48 (92.3)	16 (94.1)	32 (91.4)	
CK-MB, ng/L, median (IQR)	1.18 (0.79–2.01)	0.91 (0.66–1.98)	1.35 (0.94–2.13)	0.499
≤ 5—*n* (%)	50 (96.2)	15 (88.2)	35 (100.0)	0.103
> 5—*n* (%)	2 (3.8)	2 (11.8)	0	
BNP, pg/L, median (IQR)	323 (204–589)	303 (188–457)	358 (204–607)	0.693
≤ 450—*n* (%)	24 (46.2)	3 (17.6)	21 (60.0)	0.004
> 450—*n* (%)	28 (53.8)	14 (82.4)	14 (40.0)	
CRP, mg/L, median (IQR)	73 (46–120)	73 (41–117)	73 (47–121)	0.976
≤ 5—*n* (%)	2 (3.8)	1 (5.9)	1 (2.9)	1.000
> 5—*n* (%)	50 (96.2)	16 (94.1)	34 (97.1)	
PCT, ng/mL, median (IQR)	0.09 (0.05–0.23)	0.09 (0.07–0.23)	0.10 (0.05–0.24)	0.435
< 0.1—*n* (%)	28 (53.8)	11 (64.7)	17 (48.6)	0.274
≥ 0.1—*n* (%)	24 (46.2)	6 (35.3)	18 (51.4)	
TNI, ng/L, median (IQR)	0.01 (0.01–0.01)	0.01 (0.01–0.02)	0.01 (0.01–0.01)	0.682
≤ 0.04—*n* (%)	51 (98.1)	17 (100.0)	34 (97.1)	1.000
> 0.04—*n* (%)	1 (1.9)	0	1 (2.9)	
PT, second, median (IQR)	12.3 (11.7–12.8)	12.2 (11.65–12.85)	12.3 (11.7–12.8)	0.908
9–13—*n* (%)	45 (86.5)	14 (82.4)	31 (88.6)	0.855
≥ 13—*n* (%)	7 (13.5)	3 (17.6)	4 (11.4)	
D-dimer, mg/L, median (IQR)	1.9 (1.18–4.18)	1.46 (0.86–3.38)	2.35 (1.21–5.05)	0.214
≤ 0.55—*n* (%)	3 (5.8)	1 (5.9)	2 (5.7)	1.000
> 0.55—*n* (%)	49 (94.2)	16 (94.1)	33 (94.3)	

COPD = chronic obstructive pulmonary disease; PaO_2_/FiO_2_ = partial pressure of arterial oxygen to fraction of inspired oxygen ratio; PSI = Pneumonia Severity Index; NEWS2 = National Early Warning Score 2; SBP = systolic blood pressure; DBP = diastolic blood pressure; WBC = white blood cell; ALT = alanine transaminase; AST = aspartate aminotransferase; LDH = lactate dehydrogenase; CK-MB = creatine kinase myocardial band; BNP = B-type natriuretic peptide; CRP = C-reactive protein; PCT = procalcitonin; TNI = troponin I; PT = prothrombin time.

There were no significant differences between the position intervention group and standard care group in terms of baseline demographics, time to admission, oxygenation status, severity indices, and laboratory markers (Table 1). There were also no significant differences for respiratory support, antivirals, antibiotics, anticoagulants, and glucocorticoids (and duration) between the two groups (Table 2).

**Table 2.** Treatment of patients after enrollment.

Characteristics	Total (*n* = 52)	Position intervention (*n* = 17)	Standard care (*n* = 35)	*P* value
Respiratory support during study, *n* (%)				0.916
Nasal cannula and mask	37 (71.2)	13 (76.5)	24 (68.6)	
High flow ventilation	11 (21.2)	3 (17.6)	8 (22.9)	
Non-invasive mechanical ventilation	3 (5.8)	1 (5.9)	2 (5.7)	
Invasive mechanical ventilation	1 (1.9)	0 (0.0)	1 (2.9)	
Treatment				
Antivirals, *n* (%)	50 (100)	17 (100)	35 (100)	-
Antibiotics, *n* (%)	44 (84.6)	13 (76.5)	31 (88.6)	0.469
Anticoagulants, *n* (%)	10 (19.2)	2 (11.8)	8 (22.9)	0.564
Glucocorticoids, *n* (%)	34 (65.4)	12 (70.6)	22 (62.9)	0.583
Glucocorticoid duration, d, median (IQR)	3 (0–6)	3 (0–6)	3 (0–6)	0.520
CT absorption > 30%, *n* (%)				<0.001
No	25 (48.1)	1 (5.9)	24 (68.6)	
Yes	27 (51.9)	16 (94.1)	11 (31.4)	
NEWS2 (d 7), median (IQR)	6 (5–8)	5 (5–5)	6 (5–8)	0.002
1–4	10 (19.2)	6 (35.3)	4 (11.4)	0.009
5–6	22 (42.3)	9 (52.9)	13 (37.1)	
≥ 7	20 (38.5)	2 (11.8)	18 (51.4)	
NEWS2 (d 14), median (IQR)	4 (3–6)	3 (2–3)	5 (4–7)	<0.001
1–4	31 (62.0)	15 (88.2)	16 (48.5)	0.007
5–6	10 (20.0)	0 (0)	10 (30.3)	
≥ 7	9 (18.0)	2 (11.8)	7 (21.2)	
Rate of intubation avoidance, *n* (%)	51 (98)	17 (100)	34 (97.1)	1.000
Time to clinical improvement, d, median (IQR)	33 (27–41)	31 (25–40)	35 (28–42)	0.234
Time to virus shedding, d, median (IQR)	26 (19–33)	30 (20–36)	25 (18–31)	0.315
Length of hospital stay, d, median (IQR)	35 (28–43)	35 (27–52)	35 (28–42)	0.914
Adverse events, *n* (%)	0 (0)	0 (0)	0 (0)	

NEWS2 = National Early Warning Score 2.

### Outcome measures

Of 17 patients receiving position intervention, 11 had prone positioning throughout the study and six changed to lateral positioning.

The trends of changes for SpO_2_/FiO_2_, ROX index, and Borg scale with time grouped by position intervention (prone or lateral) and standard care are shown in Supplementary Fig. E1. Compared to the standard care group, there was a gradual increase in both SpO_2_/FiO_2_ and ROX index with time that was mirrored by a significant decrease of Borg scale in the prone or lateral positioning group. Nevertheless, no significant difference between prone and lateral positioning groups was observed for these parameters.

**Figure 2.**
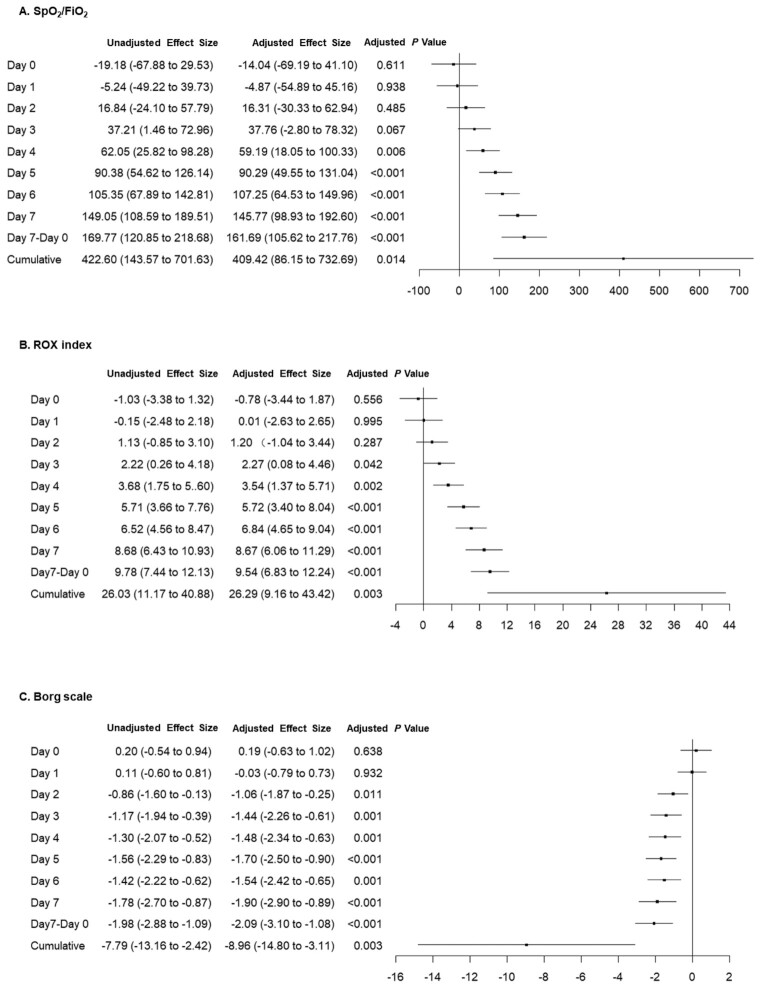
Forest plots showing between-group mean difference of position intervention vs. standard care. (A) Pulse oximetry to fraction of inspired oxygen (SpO_2_/FiO_2_). (B) Respiratory rate-Oxygenation (ROX) index. (C) Borg scale. Data are expressed as mean (95% CI).

Treatment effects of position intervention on oxygenation improvement are expressed as mean between-group difference (95% CI) and demonstrated by forest plots (Fig. 2). The mean difference of SpO_2_/FiO_2_ started to increase significantly from day 4 with a cumulative value of 409 (95% CI 86–733) after receiving position intervention for 7 days (Fig. 2A). This was accompanied by significantly increased ROX index (Fig. 2B) and decreased Borg scale (Fig. 2C) from day 3 with cumulative values of 26 (95% CI 9–43) and −9 (95% CI −15 to −3), respectively. All these findings are suggestive of early position intervention effectively improving oxygenation and reducing dyspnea as compared to standard care.

**Figure 3.**
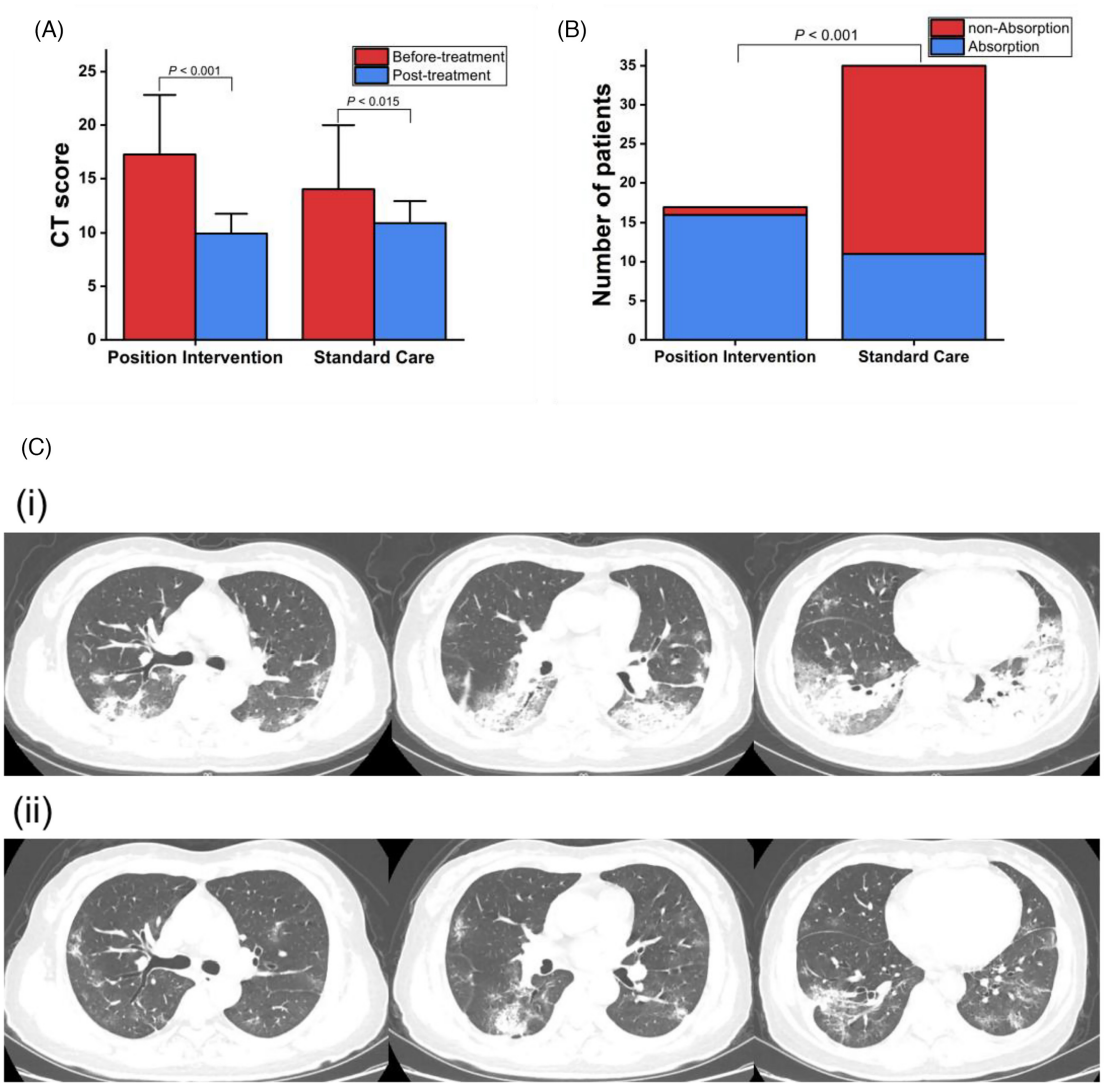
Chest CT score and changes before and after treatment. (A) Chest CT score. (B) Number of patients with lung lesion absorption > 30%. (C) Chest CT images of a 52-year-old patient with COVID-19: (i) Chest CT images obtained on 11 February 2020 show predominantly bilateral posterior consolidation. (ii) Chest CT images obtained on 20 February 2020 show the absorption of consolidation after prone position treatment from 13 February to 20 February 2020.

Chest imaging showed that the CT score for lung lesions was significantly reduced in both groups (Fig. 3A), with position intervention group: 9.9 ± 1.9, after vs. 17.2 ± 5.6, before (*P* < 0.001) and standard care group: 10.9 ± 2, after vs. 14 ± 6, before (*P* < 0.015). In corroboration with improved oxygenation, early position intervention was also associated with more patients having apparent lung lesion absorption (16/17, 94% vs. 11/35, 31%, *P* < 0.001; Fig. 3B and Table 2). Representative chest CT images of a patient who underwent prone positioning are presented in Fig. 3C.

**Figure 4.**
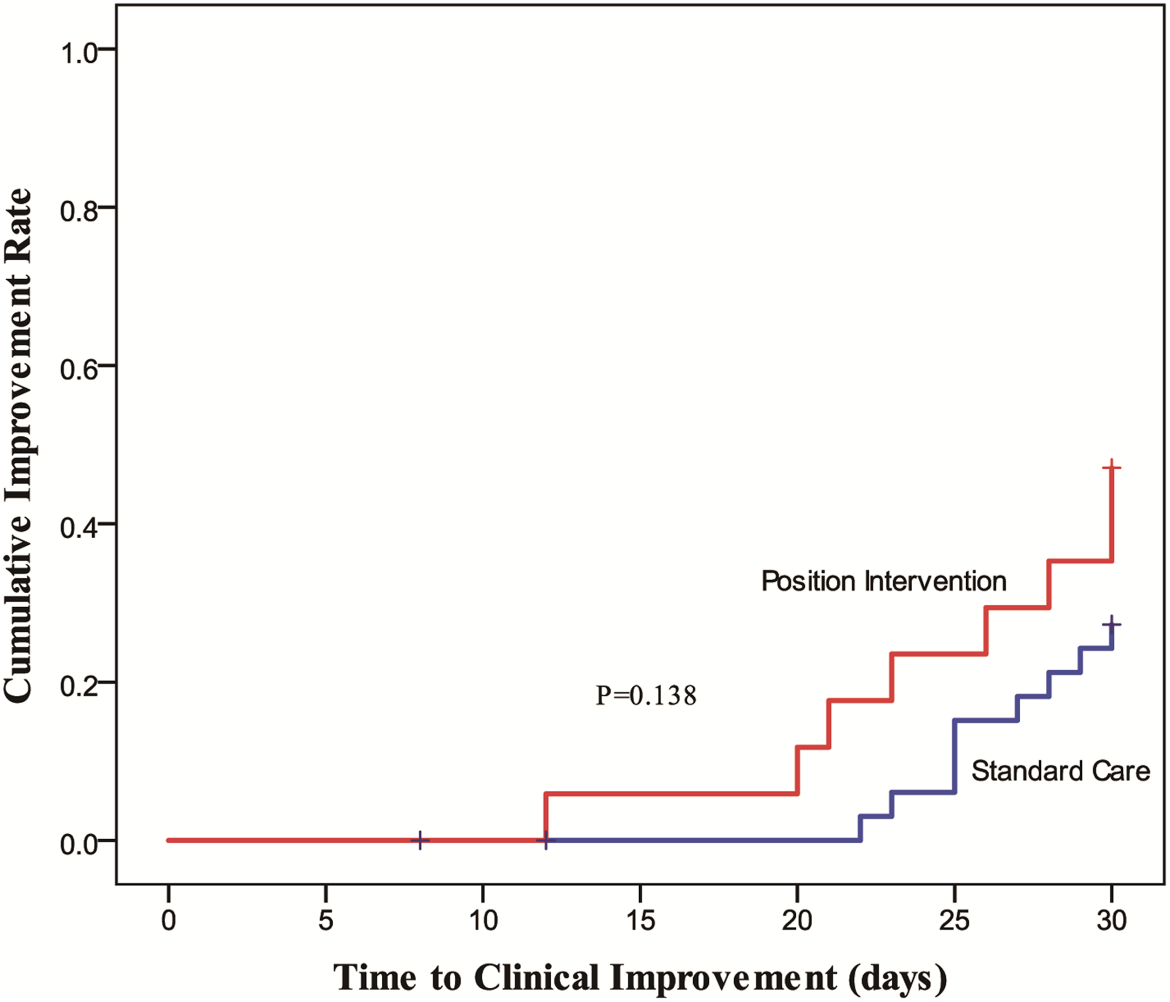
Time to clinical improvement.

The proportion of patients with high NEWS2 (≥ 7) in the position intervention group was significantly lower on days 7 (11.8% vs. 51.4%, *P* = 0.009) and 14 (11,8% vs. 21.2%, *P* = 0.007) than that in the standard care group (Table 2). There was a trend toward shortened time to clinical improvement in the position intervention group over the standard care group, albeit with no statistical significance (*P* = 0.138) (Fig. 4).

No significant differences were observed in rate of intubation avoidance, virus shedding, and length of hospital stay between the two groups (Table 2). No adverse events occurred during the study.

Results for clinical outcomes were unaltered between the two groups when intention-to-treat analysis was performed (Supplementary Fig. E2 and Table E4–5).

## Discussion

This was the first study to evaluate the efficacy and safety of early use of prone or lateral positioning on awake patients with severe COVID-19 receiving non-mechanical ventilation. Our findings indicate that position intervention significantly improved oxygenation, along with relieving subjective dyspnea and facilitating absorption of lung lesions shown in chest images. The position intervention reduced the proportion of patients with high NEWS2 on days 7 and 14, and there was a trend toward faster time to clinical improvement, but did not correlate with virus shedding, which may have influence on the length of hospital stay. Hence , the time to virus shedding and length of hospital stay showed no difference between the two groups (*P* > 0.05). The prone or lateral positioning was safely performed and well tolerated by our patients.

The main reason for ICU admission of patients with COVID-19 is ARDS, with an incidence of 6.2% in the Chinese population^3^ and 9% in Lombardy, Italy,^4^ during the pandemic period with mortalities of 51% and 26%, respectively. Our chest CT images of patients with severe COVID-19 predominantly showed exudation and consolidation near the dorsal distribution in the peripheral lung.^20^ These imaging findings are in accordance with post-mortem pathological appearances of the lungs from deceased patients with COVID-19, in that the alveolar septa were congested and edematous along with serous fibrin exudate and hyaline membrane seen in the alveolar cavity, typical signs of ARDS.^14^ The admission features of patients with severe COVID-19 were similar to those who were managed in the ICU settings in China. These patients were older and had longer time from onset to hospital admission, more comorbid hypertension, diabetes, cardiovascular and chronic lung diseases, higher platelets and D-dimer, and lower lymphocytes compared to patients with non-severe disease who were not admitted to the ICU (Supplementary Tables E1–3).

Many patients with severe COVID-19 will rapidly progress to refractory hypoxia, severe ARDS, an irreversible stage in which invasive mechanical ventilation is inevitable. Therefore, we believe early intervention may protect against patients with severe COVID-19 transitioning to the critical stage. Prone positioning during invasive mechanical ventilation for ARDS has been extensively studied, and it has been reported that improved oxygenation and lung recruitment and may decrease mortality.^23^ Nevertheless, early prone positioning is recommended for sedated patients with a threshold PaO_2_/FiO_2_ < 150 mmHg, the feasibility and timing of its application has never been appraised in awake patients with severe COVID-19. In our study, we had 59% (10/17) of patients with admission PaO_2_/FiO_2_ < 150 mmHg in the position intervention group who had faster oxygenation improvement compared with those in the standard care group (63%, 22/35). These findings imply that early position intervention in these patients may protect against progression to severe ARDS and help to avoid the need for intubation. A recent meta-analysis showed that longer prone positioning time (≥ 12 hours) was associated with lower mortality in patients with moderate to severe ARDS.^9^ However, as it was difficult for our patients to hold the unusual position for 12 hours, a 4-hour positioning procedure was adopted. This procedure appeared to be well tolerated by 17 out of 20 patients, even in patients with comorbid cardiovascular and lung diseases. The reasons for three patients dropping out were mainly subjective noncompliance rather than actual intolerance or disease severity. Patient education and encouragement may further improve tolerability and reduce nursing burdens, of particular benefit for scarce ICU resources during this global pandemic.^24^

In this study, we used the oxygen saturation index (SpO_2_/FiO_2_) to evaluate the oxygenation levels of patients. SpO_2_/FiO_2_ is a reliable, non-invasive surrogate marker for PaO_2_/FiO_2_,^25^,^26^ and is more practical for continuous oxygenation monitoring in awake patients with COVID-19. ROX index was calculated by SpO_2_/FiO_2_ to RR and was reported to have an additive effect on the accuracy for discriminating between success and failure in patients who received high-flow nasal cannula oxygen therapy.^27,28^ Here we used the ROX index to help assess clinical improvement or deterioration. The Borg scale measures breathlessness with a score of 0 to 10.^16^ It is easy to use and add a layer of reliability for the improved oxygenation determined by SpO_2_/FiO_2_ and ROX index in the position intervention group. The improvement in oxygenation parameters was also simultaneously associated with accelerated lung lesion absorption. Previous studies have investigated the mechanisms of position intervention in improving oxygenation: gravitational influence results in increased perfusion to the well-ventilated lung, which could enhance the ventilation-perfusion mismatch^29^; prone positioning promotes the re-expansion of the collapsed alveoli and improves oxygenation, which is in turn conducive to elimination of secretions.^30^ These mechanisms may also explain the effect of prone position on the clinical improvement of patients with severe COVID-19 in our study.

Patients with early prone position intervention had a lower NEWS2 on days 7 and 14, and appeared to have a shortened time to clinical improvement. All the above indicates that position intervention might reduce the risk of COVID-19 progression. Although no significant difference was detected in avoidance of mechanical ventilation between the two groups because of small sample sizes, none of the patients in the position intervention group required invasive mechanical ventilation. These results were similar to a previous study, in which it was found that patients with moderate ARDS with an initial SpO_2_ > 95% on non-invasive ventilation may benefit from early prone positioning and avoid the need for invasive ventilation.^23^

Interpreting the results of this study is limited by the small sample size, lack of randomization, and drop-out of patients from the intervention group. Nevertheless, the combination of oxygenation parameters (SpO_2_/FiO_2_, ROX, and Borg scale) were observed to be consistently improved by the position intervention, and this was also strongly supported by improved imaging of lung lesion abortion. To minimize potential bias for not being randomized, we set up the same protocols for daily management and the same criteria for imaging and discharge. Moreover, the intention-to-treat analysis did not change the overall results obtained.

In conclusion, this was the first study demonstrating that early prone or lateral positioning could improve clinical manifestation and radiological features in non-invasively ventilated patients with severe COVID-19. Because position intervention is simple, inexpensive, and safe, it may be adopted as a regular procedure for the early management of patients with severe COVID-19 without invasive ventilation. However, based on the study limitations (small number of patients, nonrandomized trial), a randomized controlled trial is recommended to confirm this hypothesis.

## Supplementary Material

pbaa034_Supplemental_File
