# Supplementary material for: Efficacy of early prone or lateral positioning in patients with severe COVID-19: a single-center prospective cohort
Source: Precis Clin Med. 2020 Sep 28;3(4):260–71. doi: 10.1093/pcmedi/pbaa034 (PMC7543626; doi:10.1093/pcmedi/pbaa034)
Supplement: pbaa034_Supplemental_File [file pbaa034_supplemental_file.doc]

**Supplementary Figures and Tables**

**Supplementary Figure Legends**

**Supplementary Figure E1.** Determination of SpO_2_/FiO_2_, ROX index, and Borg scale. Daily measurement of oxygenation parameters from ICU admission to 7 days.

**Supplementary Figure E2.** Forest plots of intention-to-treat analysis showing between-group mean difference of position intervention vs. standard care. (A) Pulse oximetry to fraction of inspired oxygen (SpO2/FiO2). (B) Respiratory rate-Oxygenation (ROX) index. (C) Borg scale. Data are expressed as mean (95% CI).

**Supplementary Figure E1.**

**
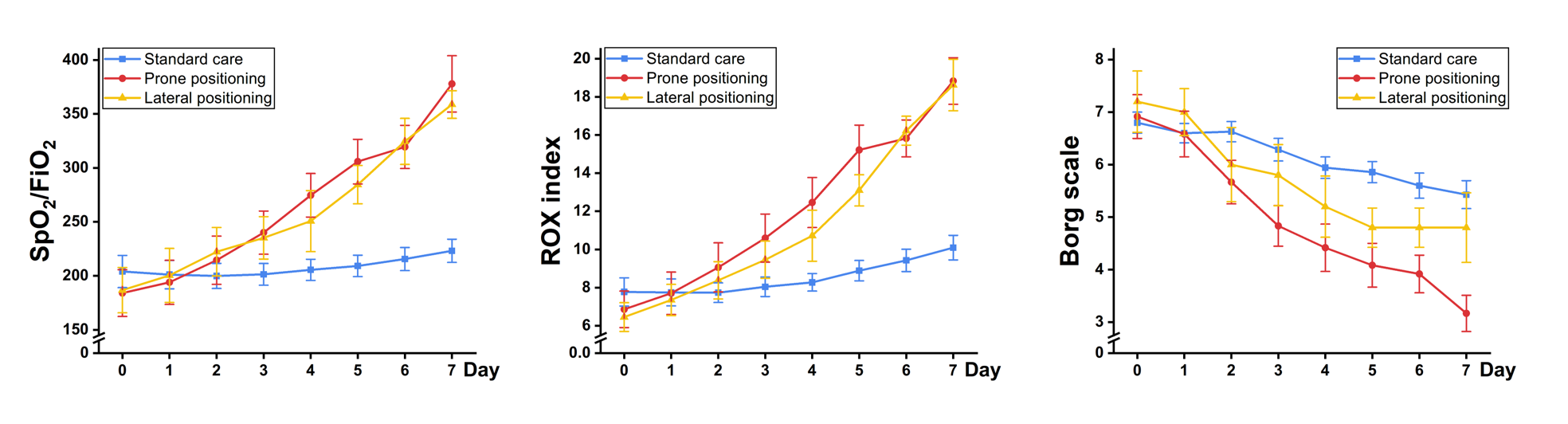
**

**S
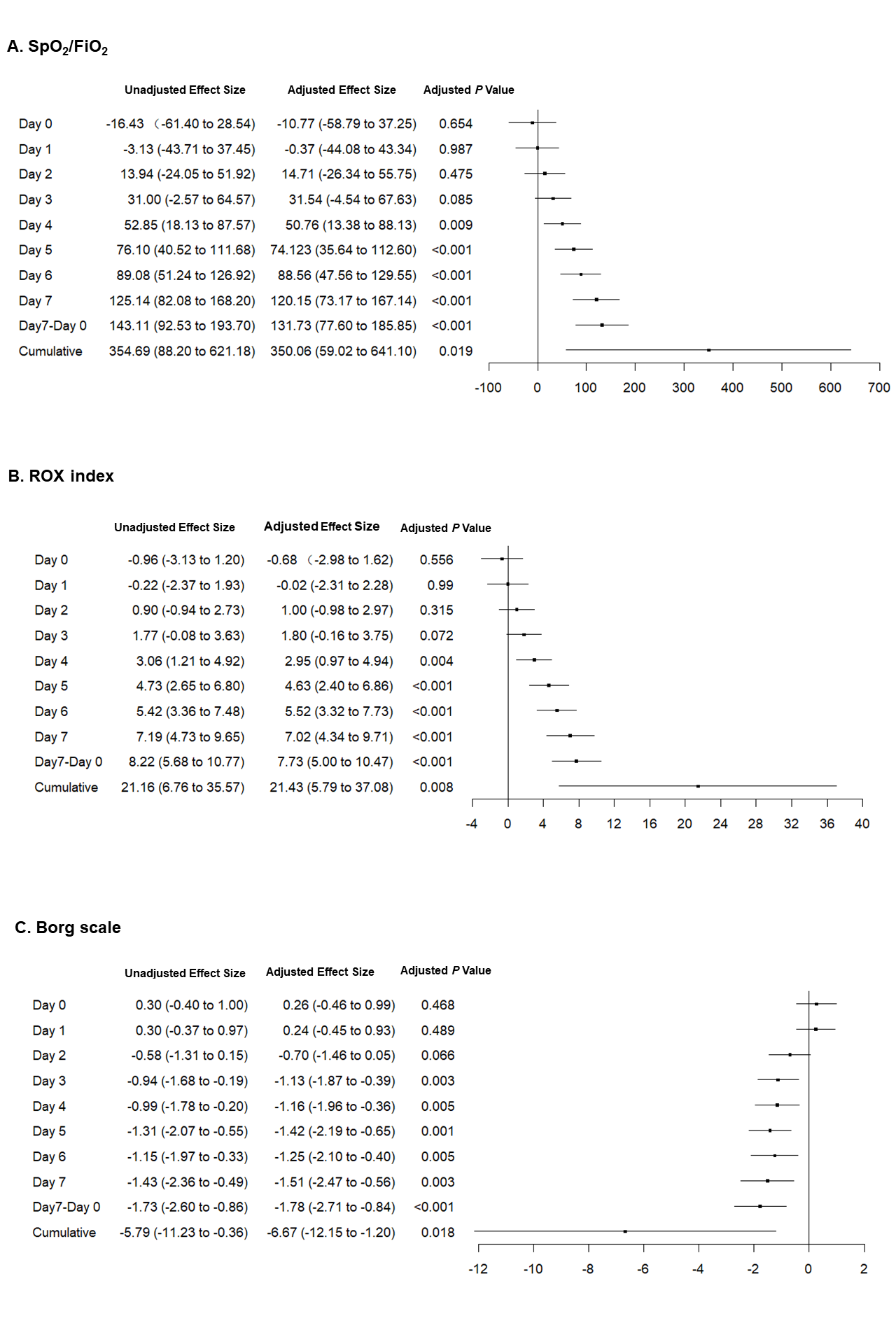
upplementary Figure E2.**

**Supplementary Table E1A.** Study Design and Quality Assessment of Included Study

| **Author** | **Country** | **Region** | **Study design** | **Center** | **Study period** | **Patient enrolment type** | **Study groups** | **Quality (NOS)** |
| --- | --- | --- | --- | --- | --- | --- | --- | --- |
| Wang et al. | China | Wuhan | Retrospective case series | Single | Jan 1 2020 to Jan 28 2020 | Consecutive | ICU vs. non-ICU | 5 |
| Guan *et al*. | China | Mainland | Retrospective observational cohort | Multiple | Until Jan 29 2020 | Consecutive | Severe vs. non-severe | 6 |
| Li *et al.* | China | Wuhan | Retrospective case series | Single | Jan 26 2020 to Feb 5 2020 | Consecutive | Severe vs. non-severe | 6 |
| Feng *et al.* | China | Wuhan, Shanghai, Anhui | Retrospective cohort | Multiple | Jan 1 2020 to Feb 15 2020 | Consecutive | Severe/critical  vs. non-severe | 6 |
| He *et al.* | China | Wuhan | Retrospective cohort | Single | Jan 10 2020 to Feb 13 2020 | Consecutive | Severe vs. non-severe | 5 |

*Definition of abbreviations*: NOS=Newcastle-Ottawa Scale; ICU=intensive care unit.

**Supplementary Table E1B.** Newcastle-Ottawa Scale Sub-sections for Included Studies

|  | **Selection** | | | | **Comparability** | **Outcome** | | |
| --- | --- | --- | --- | --- | --- | --- | --- | --- |
|  | Representativeness of the exposed cohort | Selection of the non-exposed cohort | Ascertainment of exposure | Demonstration that outcome of interest was not present at start of study | Comparability of cohorts on the basis of the design or analysis | Assessment of outcome | Was follow-up long enough for outcomes to occur | Adequacy of follow-up of cohorts |
| Wang et al. | ★ | ★ | ★ | ★ | ★ | - | - | - |
| Guan *et al*. | ★ | ★ | ★ | ★ | ★ | ★ | - | - |
| Li *et al.* | ★ | ★ | ★ | ★ | ★ | ★ | - | - |
| Feng *et al.* | ★ | ★ | ★ | ★ | ★ | ★ | - | - |
| He *et al.* | ★ | ★ | ★ | ★ | ★ | - | - | - |

**Supplementary Table E2. Demographic and Clinical Characteristics of the Patients at Baseline**

| **Author** | **Study group** | **Patient analyzed** | **Age, yr*** | **Gender, male,**  **n (%)** | **Time to admission*** | **Scoring tool** | **White cell count,**  **×10^9^/L*** | **Lymphocyte count,**  **×10^9^/L*** | **Platelet count,**  **× 10^9^/L*** | **D-dimer, mg/L*** |
| --- | --- | --- | --- | --- | --- | --- | --- | --- | --- | --- |
| Wang *et al*. | ICU | 36 | 66 (57-78) | 22 (61.1) | 8 (5-10) | WHO interim guidance (January 28 2020 version) | 6.6  (3.6-9.8) | 0.8  (0.5-0.9) | 142  (119-202) | 0.41  (0.19-1.32) |
|  | Non-ICU | 102 | 51 (37-62) | 53 (52.0) | 6 (3-7) |  | 4.3  (3.3-5.4) | 0.9  (0.6-1.2) | 165  (125-188) | 0.17  (0.10-0.29) |
| Guan *et al*. | Severe | 173 | 52 (40–65) | 100 (57.8) | — | WHO interim guidance | 3.7  (3-6.2) | 0.8  (0.6-1) | 138  (99-180) | — |
|  | Non-severe | 926 | 45 (34–57) | 537/923 (58.1) | — |  | 4.9  (3.8-6) | 1  (0.8-1.4) | 172  (139-212) | — |
| Li *et al.* | Severe | 269 | 65 (54-72) | 153 (56.9) | 10 (7-12) | Chinese management guideline for COVID-19 (version 5.0) | — | — | — | — |
|  | Non-severe | 279 | 56 (44-66) | 126 (45.2) | 9 (7-12) |  | — | — | — | — |
| Feng *et al*. | Severe | 54 | 58 (48-67) | 33 (61.1) | 7 (4-10) | Chinese management guideline for COVID-19 (version 6.0); CURB-65; MuLBSTA | 5.42  (3.69-8.17) | 0.78  (0.52-1.08) | 184  (138-216) | 0.89  (0.44-2.33) |
|  | Critical | 70 | 61 (49-68) | 48 (68.6) | 9 (7-13) |  | 7.19  (4.61-11.19) | 0.82  (0.49-1.08) | 181  (135-246) | 1.11  (0.51-4.00) |
|  | Non-severe | 352 | 51 (37-63) | 190 (54) | 6 (3-10) |  | 5.15  (4.17-6.54) | 1.13  (0.79-1.53) | 185  (146-238) | 0.51  (0.32-1.08) |
| He *et al*. | Severe | 69 | 61 (52-74) | 37 (53.6) | — | Chinese management guideline for COVID-19 (version 5.0) | 4.84  (3.83-7.85) | 0.76  (0.55-0.93) | 171  (138-217) | 0.95  (0.41-3.10) |
|  | Non-severe | 135 | 43 (31-53) | 42 (31.1) | — |  | 4.74  (4.04-5.76) | 1.43  (1.12-1.88) | 200  (167-261) | 0.32  (0.20-0.70) |

*Expressed as median (IQR).

**Supplementary Table E3.** Comorbid Conditions

| **Author** | **Study group** | **No. of patients** | **Diabetes** | **Hypertension** | **Cardio-vascular disease** | **Cerebro-vascular disease** | **Cancer** | **Chronic heart disease** | **Chronic lung disease** | **Chronic kidney disease** | **Chronic liver disease** |
| --- | --- | --- | --- | --- | --- | --- | --- | --- | --- | --- | --- |
| Wang *et al*. | ICU | 36 | 8 (22.2) | 21 (58.3) | 9 (25) | 6 (16.7) | 4 (11.1) | — | 3 (8.3) | 2 (5.6) | 0 |
|  | Non-ICU | 102 | 6 (5.9) | 22 (21.6) | 11 (10.8) | 1 (1.0) | 6 (5.9) | — | 1 (1.0) | 2 (2.0) | 4 (3.9) |
| Guan *et al*. | Severe | 173 | 28 (16.2) | 41 (23.7) | 10 (5.8) | 4 (2.3) | 3 (1.7) | — | 6 (3.5) | 3 (1.7) | — |
|  | Non-severe | 926 | 53 (5.7) | 124 (13.4) | 17 (1.8) | 11 (1.2) | 7 (0.8) | — | 6 (0.6) | 5 (0.5) | — |
| Li *et al.* | Severe | 269 | 52 (19.3) | 104 (38.7) | 28 (10.4) | 0 | 14/257 (5.5) | 3 (1.1) | 16 (5.9) | 6 (2.2) | 2 (0.7) |
|  | Non-severe | 279 | 31 (11.1) | 62 (2.2) | 6 (2.2) | 0 | 10/256 (3.9) | 2 (0.7) | 6 (2.1) | 4/248 (1.4) | 3 (1.1) |
| Feng *et al.* | Severe/critical | 124 | 17 (29.2) | 40 (63.5) | 17 (26.4) | 9 (21.3) | 7 (10.5) | — | 14 (21.3) | — | — |
|  | Non-severe | 352 | 32 (9.1) | 73 (20.7) | 21 (6) | 8 (2.3) | 5 (1.4) | — | 8 (2.3) | — | — |
| He *et al.* | Severe | 69 | 8 (11.6) | 26 (37.7) | 5 (7.3) | 7 (10.1) | 4 (5.8) | — | 0 | — | 0 |
|  | Non-severe | 135 | 8 (5.9) | 10 (7.4) | 0 | 1 (0.7) | 1 (0.7) | — | 2 (1.5) | — | 2 (1.5) |
| Summary | Severe | 671 | 113 (16.8) | 232 (34.6) | 69 (10.3) | 26 (3.9) | 53/659 (8) | — | 39 (5.8) | 11 (1.6) | 2 (0.3) |
|  | Non-severe | 1794 | 130 (7.2) | 291 (16.2) | 55 (3.1) | 21 (1.2) | 29/1771 (1.6) | — | 23 (1.3) | 11 (0.6) | 9 (0.5) |

**Supplementary Table E4. Intention-to-treat analysis of Baseline Characteristics**

| **Characteristics** | **Total**  **(*n = 55*)** | **Position intervention**  **(*n = 20*)** | **Standard care**  **(*n = 35*)** | ***P* value** |
| --- | --- | --- | --- | --- |
| Demographics |  |  |  |  |
| Age, yr, mean (SD) | 63±12 | 60 ± 12 | 64 ± 12 | 0.286 |
| Gender, male, n (%) | 33 (60.0) | 12 (60.0) | 21 (60.0) | 1.000 |
| Overall comorbidity, n (%) | 35 (63.6) | 12 (60.0) | 23 (65.7) | 0.672 |
| Hypertension, n (%) | 17 (30.9) | 7 (35.0) | 10 (28.6) | 0.620 |
| Diabetes, n (%)  Coronary heart disease, n (%)  COPD, n (%) | 12 (21.8)  8 (14.5)  6 (10.9) | 5 (25.0)  4 (20.0)  3 (15.0) | 7 (20.0)  4 (11.4)  3 (8.6) | 0.926  0.638  0.657 |
| Time to admission, d, median (IQR) | 10 (7-14) | 10 (7-12.8) | 10 (7-15) | 0.951 |
| Oxygenation status and severity |  |  |  |  |
| Respiratory rate, breaths/min, mean (SD) | 24±5 | 23 ± 4 | 26 ± 5 | 0.439 |
| PaO_2_/FiO_2_, mmHg, mean (SD) | 139±53 | 147 ± 51 | 128 ± 60 | 0.390 |
| PSI, mean (SD) | 99±20 | 100 ± 23 | 98 ± 17 | 0.739 |
| NEWS2 | 8 (7-9) | 8 (7-9) | 8 (7-9) | 0.720 |
| Laboratory indices |  |  |  |  |
| Hemoglobin, g/L | 127 (117-138) | 128 (120-131) | 127 (110-139) | 0.707 |
| WBC count, ×10^9^/L, median (IQR) | 7.06 (5.72-10.36) | 7.08 (5.79-9.90) | 7.04 (5.65-11.23) | 0.780 |
| Lymphocyte count, ×10^9^/L, median (IQR) | 0.67 (0.5-0.90) | 0.66 (0.51-0.83) | 0.68 (0.48-0.95) | 0.806 |
| Platelet count, ×10^9^/L, median (IQR) | 221 (167-289) | 179 (162-261) | 251 (167-313) | 0.032 |
| ALT, U/L, median (IQR) | 36 (23-62) | 32 (22-44) | 45 (23-65.75) | 0.436 |
| AST, U/L, median (IQR) | 33 (27-46) | 28 (23-40) | 37 (30-48) | 0.126 |
| Creatine, μmol/ L, median (IQR) | 61 (48-72) | 58 (46-77) | 63 (48-71) | 0.868 |
| LDH, U/L, median (IQR) | 378 (304-479) | 394 (306-477) | 366 (302-522) | 0.680 |
| CK-MB, ng/L, median (IQR) | 1.19 (0.78-2.27) | 0.96 (0.67-2.34) | 1.35 (0.94-2.13) | 0.192 |
| BNP, pg/L, median (IQR) | 320 (198-565) | 320 (171-468) | 358 (204-607) | 0.287 |
| CRP, mg/L, median (IQR) | 73 (46-122) | 72 (39-122) | 73 (47-121) | 0.909 |
| PCT, ng/mL, median (IQR) | 0.09 (0.05-0.22) | 0.08 (0.06-0.15) | 0.10 (0.05-0.24) | 0.495 |
| TNI, ng/L, median (IQR) | 0.01 (0.01-0.01) | 0.01 (0.01-0.03) | 0.01 (0.01-0.01) | 0.252 |
| PT, second, median (IQR) | 12.2 (11.6-12.8) | 12.0 (11.5-12.7) | 12.3 (11.7-12.8) | 0.749 |
| D-dimer, mg/L, median (IQR) | 1.9 (1.14-4.64) | 1.54 (0.80-3.93) | 2.35 (1.21-5.05) | 0.100 |

Definition of abbreviations: COPD=Chronic obstructive pulmonary disease; PaO2/FiO2= partial pressure of arterial oxygen to fraction of inspired oxygen ratio; PSI=Pneumonia Severity Index; NEWS2=National Early Warning Score 2; SBP=systolic blood pressure; DBP=diastolic blood pressure; WBC=white blood cell; ALT=alanine transaminase; AST=aspartate aminotransferase; LDH=lactate dehydrogenase; CK-MB=creatine kinase myocardial band; BNP= B-type natriuretic peptide; CRP=C-reactive protein; PCT=procalcitonin; TNI=troponin I; PT=prothrombin time.

**Supplementary Table E5. Intention-to-treat analysis of Secondary Outcomes**

| **Characteristics** | Total  (*n=55*) | Position intervention  (*n = 20*) | Standard care  (*n = 35*) | *P* value |
| --- | --- | --- | --- | --- |
| Respiratory support during study, n (%) |  |  |  | 0.857 |
| Nasal cannula and mask | 40 (72.7) | 16 (80.0) | 24 (68.6) |  |
| High flow ventilation | 11 (20.0) | 3 (15.0) | 8 (22.9) |  |
| Non-invasive mechanical ventilation | 3 (5.5) | 1 (5.0) | 2 (5.7) |  |
| Invasive mechanical ventilation | 1 (1.8) | 0 (0.0) | 1 (2.9) |  |
| NEWS2 (d 7), median (IQR) | 5 (5-8) | 5 (4-5) | 6 (5-8) | 0.004 |
| 1-4 | 10 (18.2) | 6 (30.0) | 4 (11.4) | 0.016 |
| 5-6 | 24 (43.6) | 11 (55.0) | 13 (37.1) |  |
| ≥ 7 | 21 (38.2) | 3 (15.0) | 18 (51.4) |  |
| NEWS2 (d 14), median (IQR) | 4 (3-5.75) | 3 (2-3) | 5 (4-7) | <0.001 |
| 1-4 | 34 (64.2) | 18 (90.0） | 16 (48.5) | 0.003 |
| 5-6 | 10 (18.9) | 0 | 10 (30.3) |  |
| ≥ 7 | 9 (17.0) | 2 (10.0) | 7 (21.2) |  |
| Rate of intubation avoidance, n (%) | 54 (98.2) | 20 (100) | 34 (97.1) | 1.000 |
| Time to clinical improvement, d, median (IQR) | 33 (27-41) | 32 (24-36) | 35 (28-42) | 0.183 |
| Time to virus shedding, d, median (IQR) | 27 (20-36) | 31 (20-36) | 25 (18-31) | 0.300 |
| Length of hospital stay, d, median (IQR) | 35 (28-42) | 35 (27-48) | 35.00 (28-42) | 0.868 |
| Adverse events, n (%) | 0 (0) | 0 (0) | 0 (0) | - |

Definition of abbreviations: NEWS2=National Early Warning Score 2
